# Mobile-Phase Contributions to Analyte Retention and Selectivity in Reversed-Phase Liquid Chromatography: 2. Solute-Specific Effects

**DOI:** 10.1021/acs.jpcb.5c01697

**Published:** 2025-06-16

**Authors:** Andreas Steinhoff, Alexandra Höltzel, Ulrich Tallarek

**Affiliations:** Department of Chemistry, 9377 Philipps-Universität Marburg, Hans-Meerwein-Strasse 4, Marburg 35032, Germany

## Abstract

Analyte retention in reversed-phase liquid chromatography decreases with increasing solute polarity of a compound, whereby the parameters of the water–organic solvent (W–OS) mobile phase can influence the retention order of the compounds (selectivity). Through molecular dynamics simulations in a slit-pore model of a silica-based, endcapped, C_18_ stationary phase equilibrated with W–methanol and W–acetonitrile mobile phases, we investigate how the system discriminates between small, neutral compounds with low to moderate solute polarity at the molecular level. The experimental retention behavior of the analyte ensemble was recovered by the stationary phase-averaged number of bonded-phase contacts per analyte molecule, which depends on the number of hydrophobic structural elements in a compound and its average penetration depth into the bonded-phase chains. Evasion of W contacts by the hydrophobic structural elements pushes an analyte molecule deeper into the bonded-phase chains, but only as far as allowed by its hydrogen-bond requirements, which limit the analyte density in the solvated stationary phase to locations with sufficient W density. Selectivity effects arise when mobile phase-induced changes in stationary-phase solvation alter the density limitations of a compound relative to another. Differential analyte retention results therefore from the solute-specific response to the mobile phase-controlled W density distribution in the system.

## Graphical abstract

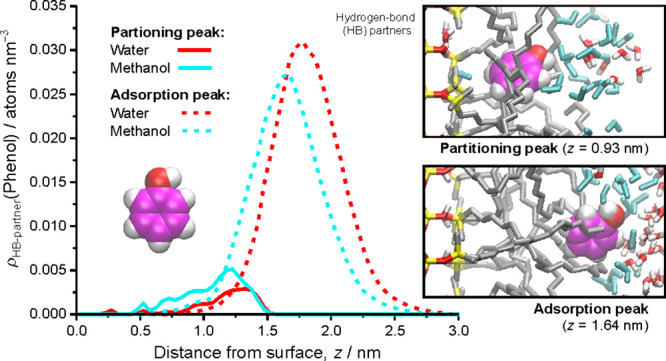

## 1 Introduction

In reversed-phase liquid chromatography (RPLC), the components of a sample (analytes) are physically separated by their sequential elution from the column with the water–organic solvent (W–OS) mobile phase.^1^ The column volume is occupied partially by the liquid mobile phase and partially by the (solvated) stationary phase, a macro–mesoporous solid, typically silica, whose surface is functionalized with the hydrophobic bonded phase, typically dimethyl-*n*-octadecylsilane (C_18_) chains. The longer an analyte resides on the column, the higher is its retention factor *k*, defined simultaneously as the time and the number distribution coefficient of analyte mass between the two compartments of the column:

$$k=\frac{t_{\mathrm{SP}}}{t_{\mathrm{MP}}}=\frac{N_{\mathrm{SP}}}{N_{\mathrm{MP}}}$$ 1

where *t*_SP_ and *t*_MP_ are the average residence times of an analyte molecule in the stationary-phase compartment and the mobile-phase compartment of the column, respectively, and *N*_SP_ and *N*_MP_ are the numbers of analyte molecules in the respective compartments at any given time. Because *t*_SP_, *t*_MP_, *N*_SP_, and *N*_MP_ are not accessible by experiment, the retention factor is in RPLC practice determined from the elution time *t*_r_ of the analyte and the dead time *t*_0_ of the column as

$$k=\frac{\left(t_{r}-t_{0}\right)}{t_{0}}=\frac{t_{r}}{t_{0}}-1$$ 2

The degree of separation between two consecutively eluting compounds with retention factors *k*_1_ and *k*_2_ is expressed as the selectivity factor α, defined as

$$\alpha =\frac{k_{2}}{k_{1}},\left(k_{2}\geq k_{1},\mathrm{so}\;\mathrm{that}\;\alpha \geq 1\right)$$ 3

The experimental retention data (Figure 1) obtained for an ensemble of six, structurally closely related, aromatic hydrocarbon analytes (Table 1) under standard separation conditions for small, neutral compounds, that is, on a silica-based, endcapped, C_18_ column with W–methanol (MeOH) or W–acetonitrile (ACN) mobile phases, largely validate two mainstay empirical rules of RPLC separations: the retention factor decreases with an increasing OS volume fraction and OS eluent strength (MeOH < ACN) in the mobile phase and with increasing solute polarity of a compound. What constitutes the solute polarity of an analyte has not been definitely decided yet, but the logarithm of the *n*-octanol–W partition coefficient, log *K*_OW_, is a widely accepted measure.^4^ In turn, RPLC provides an elegant alternative to the traditional shake flask method for the determination of log *K*_OW_ values.^5^ Interestingly, the logarithm of the *n*-hexadecane–W partition coefficient, log *K*_HW_, does not predict the elution order as well as log *K*_OW_, although the C_18_ chains are much closer in chemical structure to *n*-hexadecane than *n*-octanol chains.^6^

**Figure 1:**
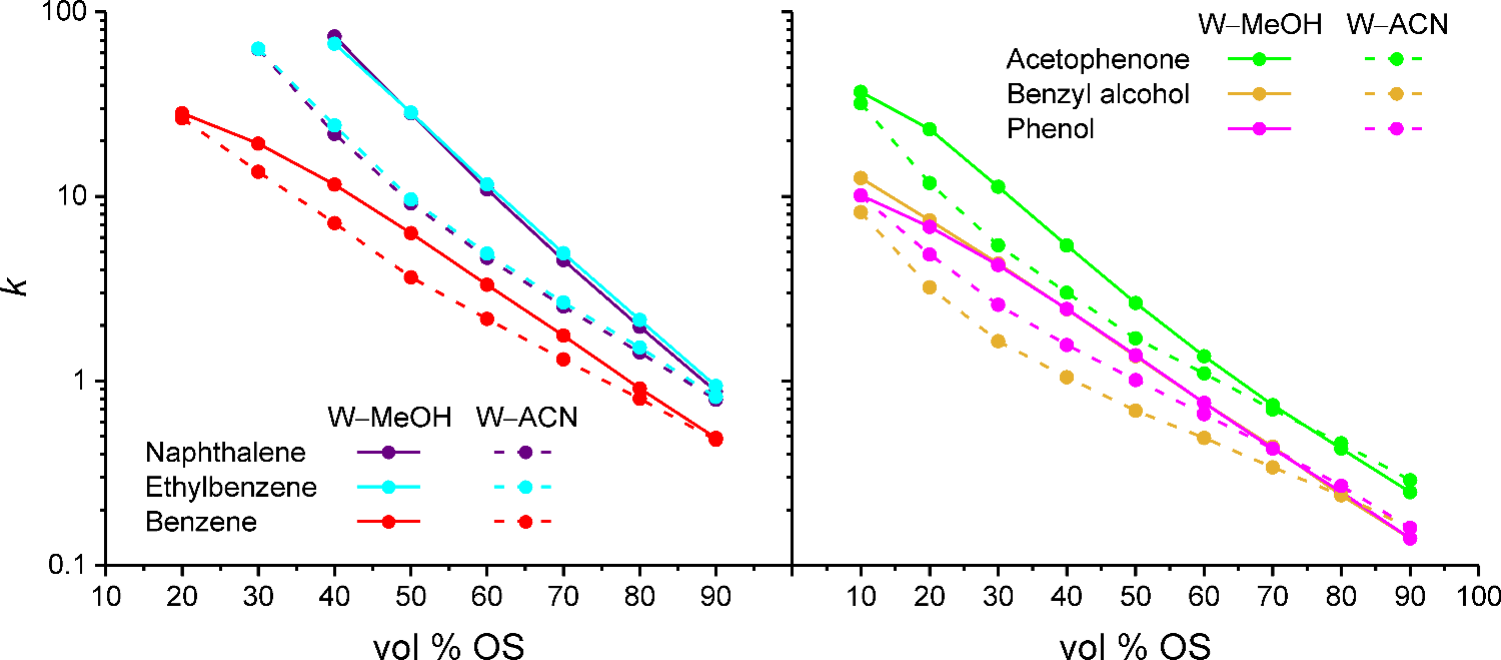
Experimental retention factors *k* obtained for apolar (left) and polar analytes (right) on a silica-based, endcapped, C_18_ column with W–MeOH and W–ACN mobile phases using uracil as a dead time marker. Experimental retention data for apolar analytes with mobile phases of low OS contents were not acquired to avoid unrealistic analysis times.^2^

**Table 1:** Solute Properties of the Analyte Ensemble

analyte	*V*_vdW_ (Å^3^)^a^	log *K*_OW_ ^b^	log *K*_HW_ ^b^	*N*(CH*_*x*_*)^c^	HB_solute–W,max_ ^d^
naphthalene	121.85	3.30	3.41	10	1.79^e^
ethylbenzene	115.78	3.15	3.20	8	1.14^e^
benzene	81.18	2.13	2.15	6	1.14^e^
acetophenone	121.93	1.58	1.14	8	2.18^e^
benzyl alcohol	107.27	1.10	–0.43	7	2.37^e^, 1.01^f^
phenol	89.97	1.46	–1.08	6	1.98^e^, 1.00^f^

a van der Waals volume calculated according to eq 4 in Zhao et al.^3^

b Experimental data reproduced with permission from Table 1 in Abraham et al.^4^

c Number of hydrophobic CH_*x*_ groups (*x* = 0, 1, 2, and 3) per solute molecule.

d Number of solute–W hydrogen bonds per solute molecule in neat W.

e With hydrogen-bond donor solvents.

f With hydrogen-bond acceptor solvents.

Figure 1 shows that a good separation of ethylbenzene and naphthalene, as indicated by selectivity factors of α > 1.1 (Table S1 in the Supporting Information), is rarely obtained with W–MeOH or W–ACN mobile phases, whereas the separation of phenol and benzyl alcohol depends strongly on the mobile-phase parameters. The log *K*_OW_ values predict phenol to be more retained than benzyl alcohol, as observed with 10–80 vol % ACN, yielding selectivity factors of α > 1.1 (Table S1). With 10–20 vol % MeOH, however, benzyl alcohol is more retained than phenol, as predicted by the log *K*_HW_ values (Table 1), and the coelution observed with >20 vol % MeOH is not predicted by the partition coefficients at all.

Apart from obvious limitations regarding the influence of the mobile-phase parameters on the observed selectivity and retention order, the solute polarity concept lacks an unequivocal correlation with the molecular structure. Considering that ethylbenzene and naphthalene differ considerably in molecular structure, it is difficult to conceive why the two compounds have similar log *K*_OW_ (and log *K*_HW_) values and why they cannot be separated on a C_18_ column, when separations of positional isomers and geometric isomers have succeeded.^7,8^ C_18_ columns are renowned for their superior methylene selectivity, which is why aromatic hydrocarbon homologues, such as benzene/toluene, toluene/ethylbenzene, etc., are used to test the efficiency of a particular column.^9^ From the perspective of methylene selectivity, benzyl alcohol should be more retained than phenol. That the opposite is observed with most mobile-phase parameters suggests that the hydrophilic OH group (with different hydrogen-bond requirements in phenol and benzyl alcohol, cf. Table 1) destroys the methylene selectivity.

The solute polarity concept is undoubtedly practical, but a liquid–liquid partitioning system cannot replicate the number and complexity of the different environments that an analyte molecule encounters in an RPLC column. Chromatographic experiments^10,11^ and molecular simulations^12–20^ have shown that the C_18_ stationary phase offers several different “sites”, whose occupation is solute-specific and modulated by the mobile-phase parameters.

To investigate the dependence of solute-specific retention from the mobile-phase parameters demonstrated by the analyte ensemble (Figure 1) at the molecular level, we first established the immediate analyte environments in the mobile-phase compartment through difference spatial distribution functions obtained from molecular dynamics (MD) simulations in bulk liquid W–OS mixtures.^2^ Figure 2 visualizes the derived general solvation pattern, according to which the hydrocarbon structural elements CH*_*x*_* (with *x* = 0, 1, 2, and 3) prefer solvation by OS molecules, whereas the functional groups OH*_n_* (with *n* = 0 and 1) prefer solvation and hydrogen-bond coordination by W molecules. The solvation pattern is maintained over a wide range of solvent ratios, reflecting that analyte molecules can shape their immediate solvation environment in the mobile phase through the rearrangement of solvent molecules in their vicinity.^2,21^

**Figure 2:**
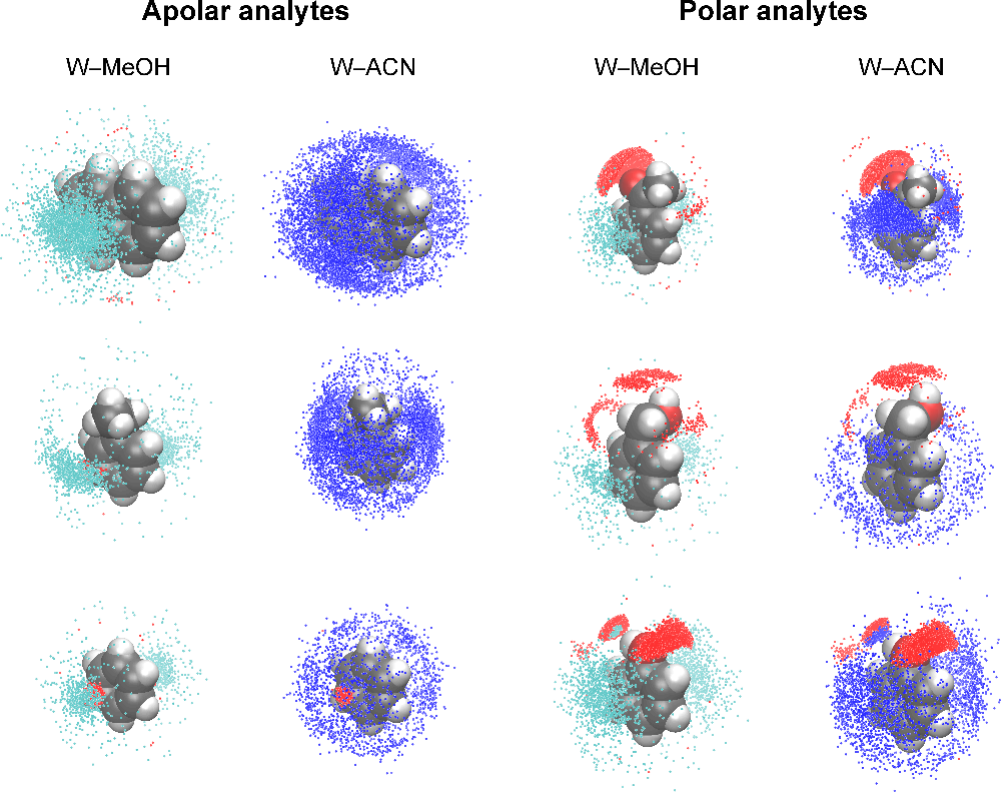
Immediate solvation environment of analyte molecules in W–MeOH and W–ACN mobile phases, shown for 50 vol % OS. Left: apolar analytes (naphthalene, ethylbenzene, and benzene from top to bottom); right: polar analytes (acetophenone, benzyl alcohol, and phenol from top to bottom). C, H, and O atoms of analyte molecules are shown as gray, white, and red balls, respectively. Colored dots indicate local excess of W over OS density (red), MeOH over W density (cyan), and ACN over W density (blue) above the threshold value (three times the bulk liquid density of the excess solvent).^2^

Assuming that the observed preferential solvation by OS molecules predicts an affinity for contact with bonded-phase groups, we proposed the number of hydrocarbon structural elements in a compound, *N*(CH*_*x*_*), as a measure for the solute’s bonded-phase affinity. Likewise, we proposed the number of solute–W hydrogen bonds per molecule in neat W, HB_solute–W,max_, as a measure for the extent to which the solute requires access to solvent molecules for hydrogen-bond coordination of its hydrophilic structural elements.^2^ All compounds of the ensemble show positive HB_solute–W,max_ values (Table 1) because the π electrons of the aromatic system form weak hydrogen bonds with donor solvents (i.e., with W and MeOH), but apolar and polar analytes remain distinguished by HB_solute–W,max_ < 2 and HB_solute–W,max_ > 2, respectively. The comparison of the solute properties in Table 1 with the retention data in Figure 1 confirms that the retention factor increases with the bonded-phase affinity and decreases with the hydrogen-bond requirements of a solute.

For exploring the immediate analyte environments in the stationary-phase compartment, we rely on MD simulations in our well-established RPLC slit-pore model,^22^ which reproduces the interfacial dynamics of a silica-based, endcapped, C_18_ stationary phase with W–OS mobile phases. Figure 3 introduces the slit-pore model with a snapshot from the simulation of phenol. The solvated stationary phase comprises the bonded-phase region, which contains the majority of the bonded-phase density, and the interfacial region, which contains the majority of the solvent density in the stationary phase.^23^ Solute partitioning into the bonded-phase region and solute adsorption to the interfacial region are the main retention events in RPLC.^12^ The bonded-phase region is characterized by low diffusivity in general, whereas the analyte diffusivity in the interfacial region profits from the presence of the OS ditch, recognizable in Figure 3 as the region around the OS density maximum.^22–25^ How the analyte density is distributed over the bonded phase and the interfacial region of the solvated stationary phase has therefore large consequences for analyte transport at the mesopore level, as recent multiscale simulations of mass transport in reconstructed macro–mesoporous column beds have shown.^26,27^

**Figure 3:**
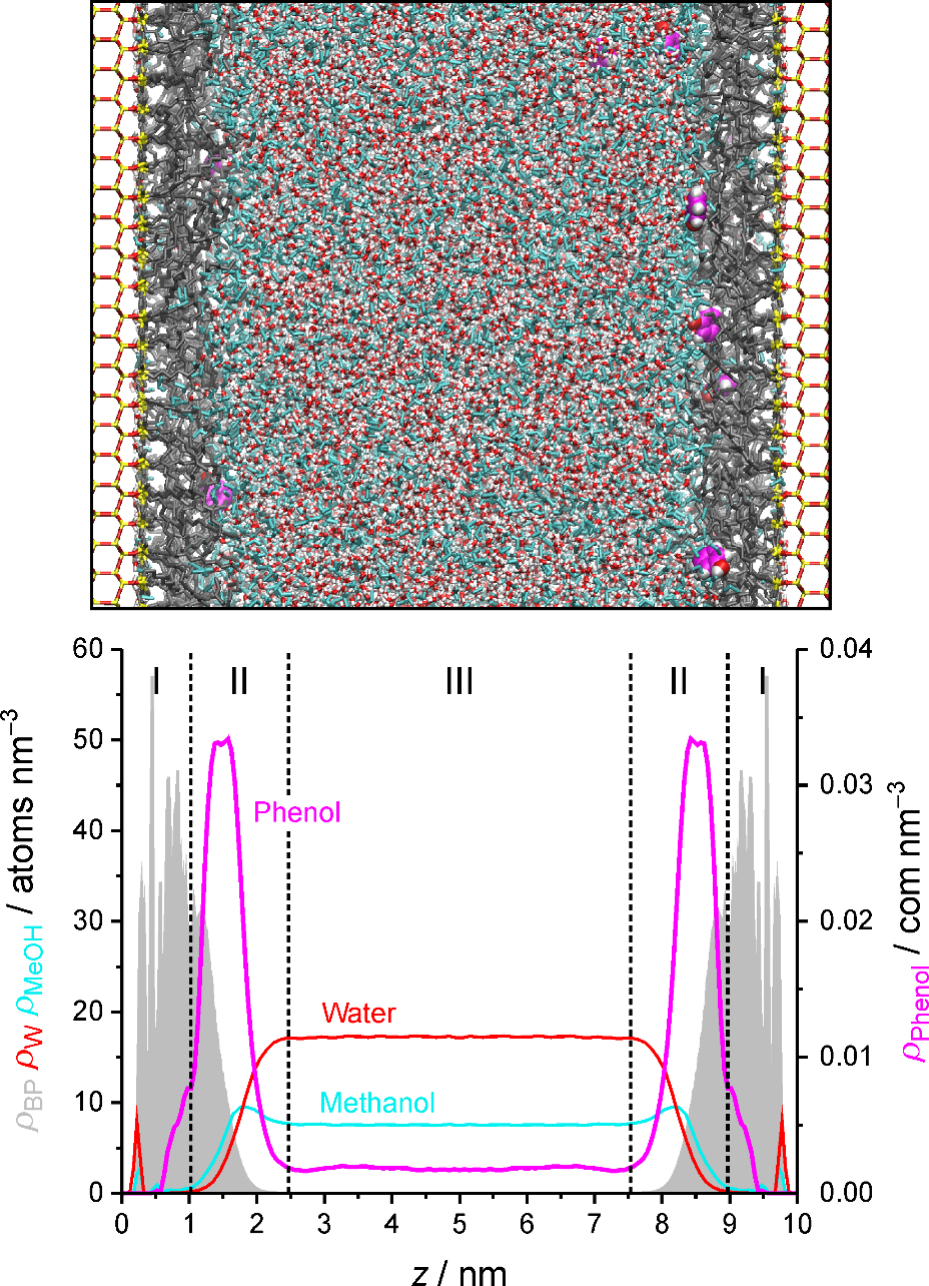
Top: Snapshot from the simulation of the analyte phenol in our RPLC slit-pore model of a silica-based, endcapped, C_18_ stationary phase, equilibrated here with a W–OS mobile phase containing 50 vol % MeOH. Yellow, red, white, and gray sticks represent Si, O, and H atoms of the silica support and bonded-phase groups, respectively. O and H atoms of W molecules are shown as red and white sticks, and MeOH molecules as cyan sticks. C, H, and O atoms of phenol molecules are shown as pink, red, and white balls. Bottom: Derived bonded-phase (gray shadow), W, MeOH, and phenol density profiles. Dashed vertical lines indicate the approximate extensions of the bonded-phase (I) and interfacial region (II) of the solvated stationary phase as well as the bulk liquid region of the pore (III).

In the first part of the study,^28^ we used benzene as the structurally simplest compound of the ensemble to identify the composition of the immediate analyte environments in the solvated stationary phase through spatially resolved contact analysis and elucidate how the mobile phase controls analyte retention in general. We showed that the number of analyte contacts with bonded-phase groups is high in the bonded-phase region and declines over the interfacial region toward the bulk liquid region. The number of bonded-phase contacts per analyte molecule, averaged over the extension of the solvated stationary phase, ⟨*C*_BP_⟩_SP_, was introduced as a measure for the retentivity of the solvated stationary phase toward the analyte. Like the retention factor, ⟨*C*_BP_⟩_SP_ decreases with increasing mobile-phase elution strength.

The correlated analysis of bonded-phase, solvent, and analyte density profiles revealed that the mobile phase controls the retentivity of the stationary phase through its solvation. Increasing the mobile-phase elution strength raises the OS density in the solvent-depleted part of the bonded-phase region (*z* = 0.4–1.05 nm, Figure 3), lowers the W density in the interfacial region, and redistributes and extends the bonded-phase density toward the bulk liquid region through various changes of the C_18_ chain conformation (cf. Table 1 and Figures 5–7 in ref 28). The retreat of W density from the interfacial region was shown to favor the occupation of analyte environments closer to the bulk liquid region, resulting in a loss of bonded-phase contacts.

The data obtained in the first part of the study strongly suggested the evasion of W contacts as the principle behind the benzene density distribution in the solvated stationary phase. However, the phenol density profile (Figure 3) already shows that the extent to which analyte molecules evade W contacts depends on solute properties, as the W-deprived bonded-phase region holds <10% of the phenol density in the stationary phase (compared to 35% of the benzene density, cf. Figure 1 in ref 28). Unlike in the mobile phase, an analyte molecule in the solvated stationary phase cannot shape its immediate environment, but it can choose only which environment it occupies. If compounds with hydrophilic functional groups occupied only environments that offer sufficient access to W molecules, as the phenol density profile suggests, then the analyte selectivity would depend on stationary-phase solvation.

In the second part of this study, we continue the MD simulations in the RPLC slit-pore model with the full analyte ensemble to answer the question of how the solvation of the C_18_ stationary phase by the W–OS mobile phase contributes to the differential retention of analyte compounds and thus to analyte selectivity. In particular, we aim to understand the coelution of naphthalene and ethylbenzene and the mobile-phase sensitivity of phenol and benzyl alcohol. By focusing on the solute-specific response to the mobile-phase parameters, we intend to find out which solute properties inform the analyte density distribution and ultimately the retention factor.

## 2 Methods

### 2.1 Simulations

For each compound of the analyte ensemble, MD simulations were carried out in an RPLC slit-pore model with W–MeOH and W–ACN mobile phases and in a bulk box with neat W (to obtain HB_solute–W,max_, Table 1). The compositional range simulated in the slit-pore model (20–90 vol % OS and 10–90 vol % OS for apolar and polar analytes, respectively) either matched or exceeded the range for which experimental retention data were available from an earlier study.^2^ Analyte trajectories in the RPLC slit-pore model were partially generated by extending trajectories from a previous MD simulation study of surface diffusion.^23^ Productive analyte trajectories, preceded by equilibration periods of 34–113 ns, were 370–1169 ns long with W–MeOH mobile phases and 398–1142 ns long with W–ACN mobile phases.

In total, the study comprised 102 simulation systems in the slit-pore model (6 compounds × 2 OS types × 8–9 OS volume fractions) and six simulation systems in the bulk box, carried out using 144 and 72 cores per system, respectively, on the high-performance computer HoreKa of the Steinbuch Center for Computing at the Karlsruhe Institute of Technology (Karlsruhe, Germany), resulting in a total of 1,500,000 core hours.

#### 2.1.1 Simulation Details

Simulations in the slit-pore model were carried out in the canonical *NVT* ensemble (constant number of particles *N*, simulation box volume *V*, and temperature *T* = 300 K) with GROMACS version 2019.6.^29,30^ The construction of the functionalized silica surface,^31^ the choice and validation of the force field parameters for the system components,^32,33^ and the simulation protocol have been described in detail earlier.^23,34^ The simulation box (*xyz* = 12.14 nm × 13.20 nm × 10.93 nm) contained a centrally positioned, plane silica slab (0.93 nm in the *z*-direction) between two solvent reservoirs. The applied periodic boundary conditions turned the system into a 10 nm wide slit pore (cf. Figure 3). The silica surface bore C_18_ chains, trimethylsilane endcapping groups, and residual OH groups at densities of 3.11, 0.93, and 3.42 μmol m^–2^, respectively. Silica surface atoms and bonded-phase groups were modeled with the force field parameters of Gulmen and Thompson and the united-atom version of the transferable potentials for phase equilibria (UA-TraPPE) for *n*-alkanes, respectively.^35,36^ The simulation box contained 10 analyte molecules of a given compound, modeled with the CHARMM general force field,^37^ and the required number of W and OS molecules to recover the targeted OS volume fraction in the bulk liquid region of the pore. W molecules in W–MeOH and W–ACN mobile phases were modeled with the TIP4P/2005 and the extended simple point charge (SPC/E) force field, respectively.^38,39^ Different W force fields were used for W–MeOH and W–ACN mobile phases to obtain the best approximation of the experimental solvent densities and self-diffusivities in the respective bulk liquid mixtures over the full range of OS volume fractions.^23,32,33^ Solvent molecules were modeled with the UA-TraPPE versions for alcohols (MeOH) and nitriles (ACN).^40,41^ The numbers of W and MeOH molecules or W and ACN molecules in the simulation box for a given OS volume fraction in the mobile phase (listed in Table S1 of ref 28) were determined in prior preparatory simulation runs that mimic the column equilibration process in RPLC practice.

Bulk box simulations were carried out for 10–20 ns in the *NpT* ensemble (constant *N*, pressure *p* = 1 bar, and *T* = 300 K) followed by 50 ns in the canonical *NVT* ensemble with GROMACS version 2020.6.^29,30^ For equilibration in the *NpT* ensemble, we used the Berendsen barostat with a coupling constant of 0.1 ps and a compressibility of 4.5 × 10^–5^ bar^–1^. The bulk box contained 10 analyte molecules of a given compound, modeled with the CHARMM general force field,^37^ and 20,000 W molecules modeled with the TIP4P/2005 force field.^38^ A preparatory evaluation had shown that the SPC/E force field generated slightly higher HB_solute–W,max_ values for the compounds than the TIP4P/2005 force field, but the difference was negligible (<5%).

For MD simulations in the *NVT* ensemble, the temperature was kept constant with the Nosé–Hoover thermostat with a coupling constant of 0.25 ps. Energy minimization was conducted with the steepest descent method before simulations were started. Initial particle velocities were randomly assigned with a Maxwell–Boltzmann distribution. Long-range electrostatic interactions were treated with the particle-mesh Ewald algorithm. A 12–6 Lennard-Jones potential was used for nonbonded interactions, and Lorentz–Berthelot combination rules were applied to treat Lennard-Jones parameters for unlike interactions. A validated cutoff radius of 1.4 nm was chosen for all interactions.^42^ Bonds were constrained using the GROMACS default algorithm LINCS (LINear Constraint Solver) and the GROMACS implementation of the SETTLE algorithm (for W molecules).

To enable the replication of the simulation systems in this study, GROMACS files for the simulation of the analyte benzene with a W–OS mobile phase containing 20 vol % MeOH or 20 vol % ACN were uploaded to the Zenodo repository. We also uploaded the configuration of the solvent-free RPLC slit pore with the force field parameters for the other five compounds of the analyte ensemble. The necessary number of W and OS molecules in the simulation box for each OS volume fraction of the W–MeOH or W–ACN mobile phase can be found in Table S1 of the Supporting Information of ref 28.

### 2.2 Data Analysis

#### 2.2.1 Determination of the Stationary-Phase Limit

Except for analyte density profiles, ρ_analyte_(*z*), which were calculated from the full analyte trajectories using a bin width of 0.05 nm, and for bulk liquid HB_solute–solvent_ data (see Section 2.2.3), the calculated properties involve the use of artificially introduced, spatial boundaries, at least in the application of the stationary-phase limit. Previous work about the function of dead time markers in RPLC has shown that a stationary-phase limit does not reveal itself at the molecular level and thus needs to be defined for the calculation of retention data.^34^ The method we developed to define the stationary-phase limit relies on the decay of analyte–bonded-phase contacts toward the bulk liquid region. The stationary-phase limit was determined as the distance from the silica surface, *z*_SP_, where an analyte molecule within an observation interval of 40 ns has, on average, less than one bonded-phase contact (see Section 2.2.2). The derived *z*_SP_ values depend on the analyte compound and the bonded-phase extension, which, in turn, depends on the OS type and OS volume fraction in the mobile phase. The *z*_SP_ values for apolar and polar analytes are listed in Tables S2 and S3 of the Supporting Information, respectively.

In the first part of this study, we demonstrated in detail how mobile phase-induced changes in the conformation of the C_18_ chains lead to an overall bonded-phase density extension toward the bulk liquid region.^28^ The *z*_SP_ values in Tables S2 and S3 do not strictly reflect the evolution of the bonded-phase extension with an increasing OS volume fraction in the mobile phase, as small fluctuations in the average positions of analyte molecules and bonded-phase groups persist throughout the trajectory, particularly around the highly mobile OS ditch, where the stationary-phase limit is located. For the same reason, completely smooth curves cannot be expected when averaged data are presented as a function of the OS volume fraction in the mobile phase.

#### 2.2.2 Calculation of Stationary Phase-Averaged Analyte Positions and Bonded-Phase Contacts

The stationary phase-averaged analyte position, ⟨*z*_analyte_⟩_SP_, was determined as the weighted average of the distance of the center-of-mass (com) of an analyte molecule from the silica surface regarding the analyte density distribution at *z* ≤ *z*_SP_. The stationary phase-averaged number of bonded-phase contacts per analyte molecule, ⟨*C*_BP_⟩_SP_, was likewise determined as the weighted average of the bonded-phase contact profile, *C*_BP_(*z*), regarding the analyte density distribution at *z* ≤ *z*_SP_, whereby *C*_BP_(*z*) gives the number of bonded-phase contacts per analyte molecule at a given distance *z* from the silica surface (cf. Figure 9 in ref 28). Bonded-phase contact profiles were obtained from 40 ns trajectories by using a bin width of 0.05 nm by counting the number of bonded-phase groups within a radius of *r* ≤ *r*_BP_.

Solute-specific *r*_BP_ values were derived from radial distribution functions (RDFs), calculated pairwise between the com of analyte molecules and the united-atom groups of the bonded phase and averaged over the vol % OS range available for a given compound and OS type. Solute-specific *r*_BP_ values were *r*_BP_ = 0.84 (naphthalene and ethylbenzene), 0.81 (acetophenone), 0.80 (benzyl alcohol), and 0.79 nm (phenol, benzene) with W–MeOH mobile phases and *r*_BP_ = 0.83 (naphthalene and ethylbenzene), 0.81 (acetophenone), 0.79 (benzyl alcohol and benzene), and 0.78 nm (phenol) with W–ACN mobile phases.

Standard deviations for ⟨*C*_BP_⟩_SP_ were between ±0.85 and ±2.03 with W–MeOH mobile phases and between ±0.78 and ±1.83 with W–ACN mobile phases.

#### 2.2.3 Calculation of Solute–Solvent Hydrogen Bonds and Hydrogen-Bond Partner Densities

Solute–solvent hydrogen bonds were determined based on a distance criterion *r* ≤ *r*_HB_, whereby the applied cutoff distances *r*_HB_ were available from earlier work.^2^ Solute-specific *r*_HB_ values for the donor and acceptor atoms of solute and solvent molecules involved in hydrogen-bond formation by the functional groups of the polar analytes are listed in Table S4. For hydrogen bonds involving π electrons, we used general cutoff values for the distance between the com of the aromatic C atoms and the H atom of W molecules (*r*_HB_ = 0.30 nm) or MeOH molecules (*r*_HB_ = 0.32 nm). Values for HB_solute–solvent_ were determined from 30 ns trajectories (functional groups) or 10 ns trajectories (π electrons) using a bin width of 0.05 nm.

Hydrogen-bond partner densities were calculated as the number densities of the O atom of W molecules, the O atom of MeOH molecules, and the N atom of ACN molecules involved in solute–solvent hydrogen bonds from 30 ns trajectories using a bin width of 0.05 nm. Hydrogen-bond partner densities for the partitioning peak and the adsorption peak were calculated based on the respective analyte position.

Standard deviations for HB_solute(π)–solvent_ were between ±0.01 and ±0.08 with W–MeOH mobile phases and between ±0.00 and ±0.13 with W–ACN mobile phases. Standard deviations for HB_solute–solvent_ involving the functional groups were between ±0.02 and ±0.12 with W–MeOH mobile phases and between ±0.02 and ±0.10 with W–ACN mobile phases.

#### 2.2.4 Determination of Solute Orientation

The analyte orientation was traced through the angle φ between the surface normal and a solute-specific, molecular vector, defined such that cos φ < 0 and cos φ > 0 indicate the silica-surface and bulk-liquid orientation, respectively, of the functional group or side chain. Probability distributions for cos φ were calculated as a function of the distance *z* from the full analyte trajectories by using a bin width of 0.05 nm for the spatial resolution and a bin width of 0.1 for cos φ. The preferential analyte orientation in the partitioning peak and the adsorption peak (Figures S1–S4) was then determined as the weighted average of the probability distribution for cos φ in the corresponding *z*-interval with respect to the analyte density distribution in this *z*-interval.

## 3 Results and Discussion

### 3.1 Bonded-Phase Contacts vs Retention Factors

We begin the presentation of our results by recalling that the stationary phase-averaged number of bonded-phase contacts per analyte molecule, ⟨*C*_BP_⟩_SP_, derived as a retentivity measure in the first part of the study,^28^ reflects at once the bonded-phase density of the immediate analyte environments and their relative population by the analyte molecules. The value of ⟨*C*_BP_⟩_SP_ decreases with increasing mobile-phase elution strength because the associated retreat of W density from the solvated stationary phase favors the population of environments closer to the bulk liquid region, corresponding to an analyte density shift toward locations of lower bonded-phase density.^28^

In Figure 4, the ⟨*C*_BP_⟩_SP_ values for the analyte ensemble are displayed intentionally in the same way as the analyte retention factors in Figure 1 to facilitate the comparison of simulation-derived and experimental data. Regarding the dependence from the mobile-phase parameters, Figure 4 shows that the ⟨*C*_BP_⟩_SP_ values generally decrease with increasing mobile-phase elution strength, as previously observed for benzene,^28^ confirming that the mobile phase modulates the retentivity of the stationary phase toward analyte compounds. Surprisingly, the ⟨*C*_BP_⟩_SP_ values also reflect the solute-specific retention behavior and thus the selectivity of the stationary phase, very well. Although the experimental retention of naphthalene is slightly underestimated by the ⟨*C*_BP_⟩_SP_ values, the retention order of the analyte ensemble observed with most mobile phases is correctly reproduced in Figure 4. Moreover, the ⟨*C*_BP_⟩_SP_ values depict the selectivity of the critical analyte pair phenol/benzyl alcohol quite faithfully, including the sensitivity to the OS type and OS volume fraction in the mobile phase.

**Figure 4:**
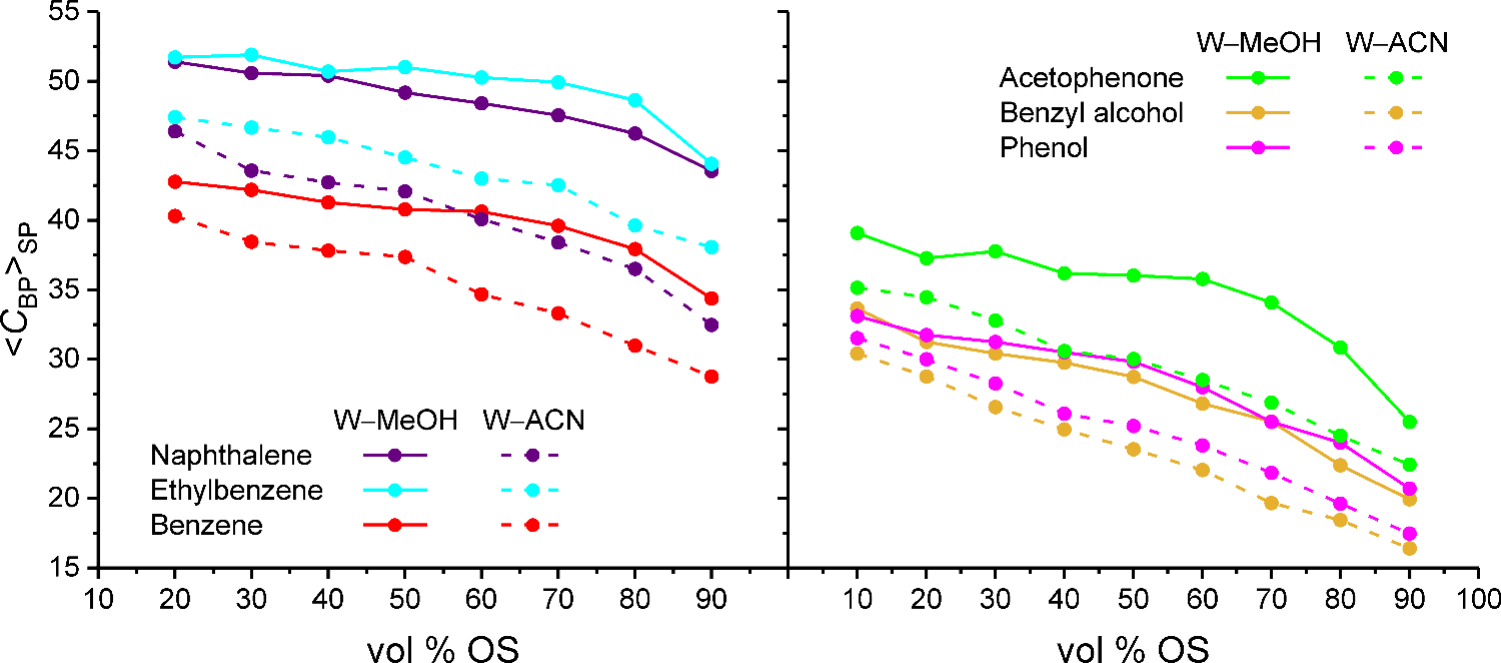
Average number of bonded-phase contacts per analyte molecule for apolar (left) and polar analytes (right) in the solvated stationary phase as a function of the mobile-phase parameters. Data for benzene reproduced with permission from ref 28. Copyright 2025 American Chemical Society.

The reproduction of the analyte retention order (cf. Figure 1) by the ⟨*C*_BP_⟩_SP_ values in Figure 4 proves an underlying assumption of chromatographic separations, namely, stronger analyte retention originates from increased analyte–stationary phase interactions. This statement may appear trivial, but that is not the case at all, considering that the retention factor quantifies only the distribution of analyte mass between the two compartments of a column (eq 1). The retention factor decreases when analyte molecules leave the stationary-phase compartment for the mobile-phase compartment (the bulk liquid region in the slit-pore model). To obtain selectivity from this process, it would suffice that analyte compounds have different affinities for the mobile phase. However, we now find that analyte compounds have different affinities for the (solvated) stationary phase as well and that this affinity can be quantified by the number of contacts between an analyte molecule and bonded-phase groups.

To estimate the mobile-phase affinity of the analyte compounds, we revisit their solvation shells in bulk liquid W–OS mixtures.^2^ Figure 5 shows the limiting linear preferential solvation values for the solutes by the OS, δ_S,OS_^0^, in W–MeOH and W–ACN mixtures.^43^ The δ_S,OS_^0^ data in Figure 5 originate from the same RDFs that underlie the immediate solvation environments (Figure 2), but the δ_S,OS_^0^ data quantify the overall OS excess in the solvation shell, whereas the immediate solvation environments display the spatial distribution of W and OS excess in the solvation shell. Between 10 and 90 vol % OS in the mobile phase, the solvation shell of every compound in the analyte ensemble holds an overall OS excess, as Figures 2 and 5 show, while W excess is confined to the functional groups or the π electrons. The predominance of hydrophobic structural elements in a compound and the associated overall OS excess in the solute solvation shell distinguish RPLC analytes from dead time markers.^34^

**Figure 5:**
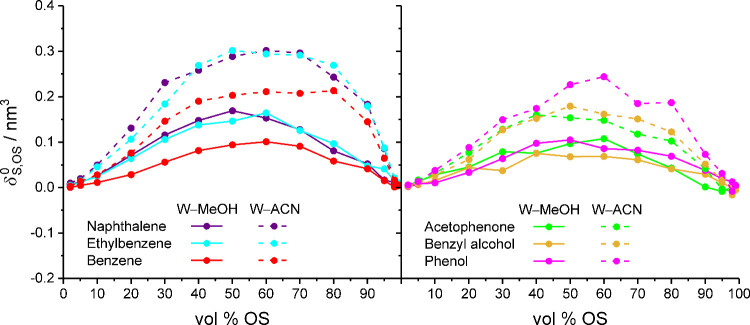
Limiting linear preferential solvation by the OS derived for apolar (left) and polar analytes (right) in bulk liquid W–MeOH or W–ACN mixtures.^2^

The comparison of Figures 1 and 5 shows that whereas the retention behavior of the apolar analytes is well-represented by the OS excess in the solute solvation shells, this applies neither to the retention behavior of the polar analytes nor to the retention gap between benzene and the polar analytes. The phenol solvation shell, for example, holds similar or more OS excess than the acetophenone solvation shell, but phenol is consistently less retained than acetophenone. Similarly, benzene and phenol hold a similar amount of OS excess in their solvation shells, but the two compounds differ considerably in retention. This means that the discrimination between the compounds based on preferential solvation by the OS (Figure 5) is weaker than the discrimination based on bonded-phase contacts (Figure 4). Moreover, the extent of preferential solvation by the OS does not reflect the experimentally observed retention order, but the ⟨*C*_BP_⟩_SP_ values do. This shows that differential analyte retention and thus selectivity in RPLC separations rely primarily on the bonded-phase affinities of the compounds.

In summary, the value of ⟨*C*_BP_⟩_SP_, which is informed by the composition and occupation of the available analyte environments in the solvated stationary phase, captures the dependence of the analyte retention factor from the mobile-phase elution strength and the solute properties. Therefore, ⟨*C*_BP_⟩_SP_ allows several interpretations relevant to analyte retention: as a measure for the retentivity of the solvated stationary phase toward an analyte, for the bonded-phase affinity of the analyte, and for the average environment of the analyte in the solvated stationary phase. Interestingly, if we compare Figures 4 and 2 and remember that the analytes maintain their bulk liquid solvation pattern over a wide range of solvent compositions, it becomes clear that the average analyte environment in the solvated stationary phase changes more than the immediate analyte environment in the bulk liquid region upon an increase in the OS volume fraction in the mobile phase. This suggests that the loss of analyte retention observed with an increasing OS volume fraction in the mobile phase originates not so much from a more favorable mobile-phase compartment than a less retentive stationary-phase compartment.

### 3.2 Analyte Density Distribution in the Solvated Stationary Phase

The reproduction of the experimentally observed retention behavior of the analyte ensemble by the ⟨*C*_BP_⟩_SP_ values (Figure 4) indicates that differential analyte retention is tied to solute-specific differences between the analyte density distributions in the solvated stationary phase. The general effect of increasing mobile-phase elution strength on the analyte density distribution (how the mobile phase controls analyte retention) was elucidated in the first part of the study, using benzene as probe molecule.^28^ In the following, we discuss mobile-phase effects only in view of selectivity.

#### 3.2.1 Solute-Specific Differences between the Analyte Density Distributions

The comparison of Figures 6 and 7 shows that solute-specific differences between the analyte density profiles are most pronounced in the bonded-phase region (*z* ≤ 1.05 nm). First, apolar analytes have more density in the bonded-phase region than polar analytes. Second, the apolar analytes have quite similar density profiles, whereas the polar analytes have highly solute-specific density profiles that differ in their sensitivity to the mobile-phase composition.

**Figure 6:**
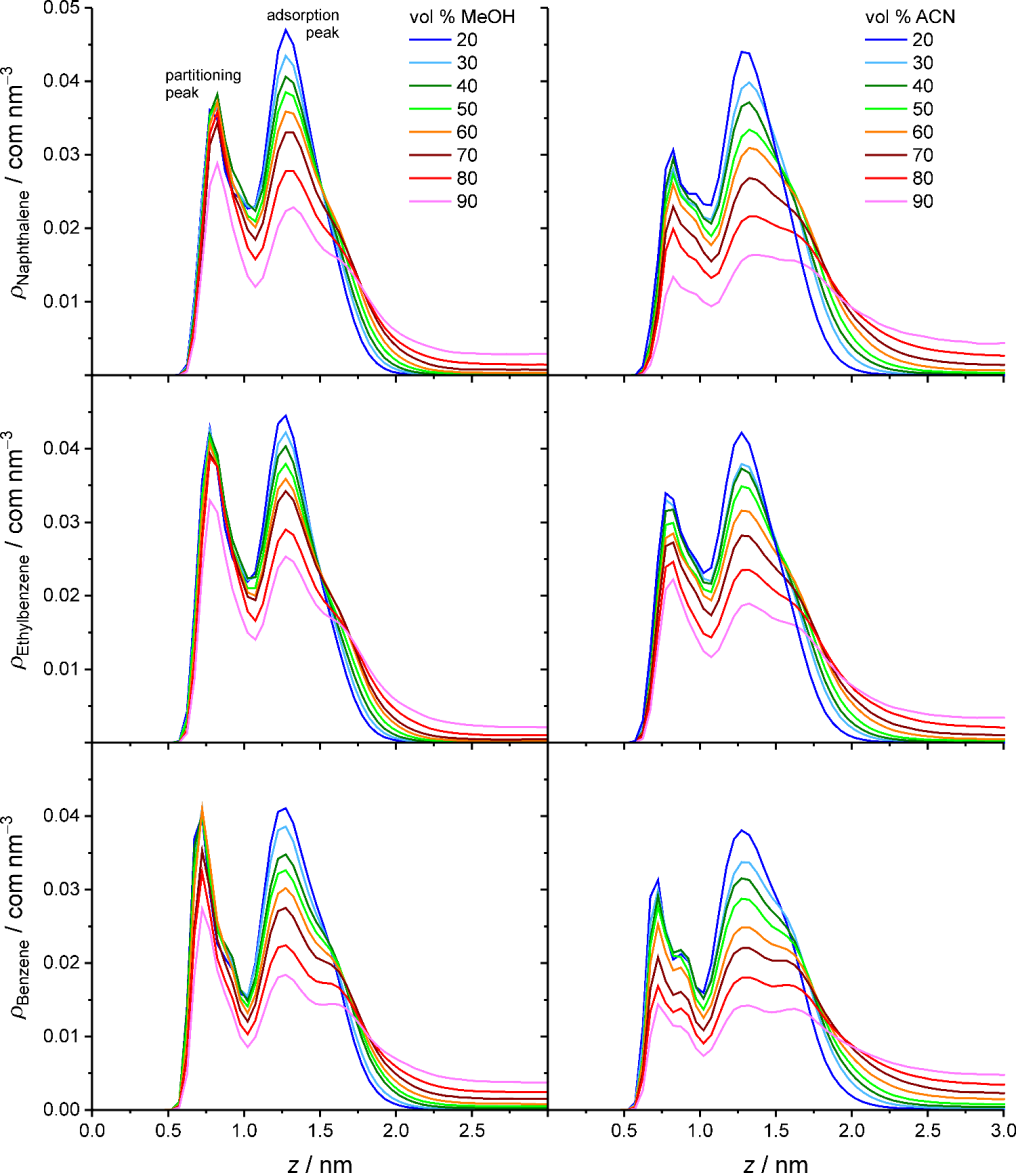
Density profiles for apolar analytes in the solvated stationary phase as a function of the mobile-phase parameters. Data for benzene reproduced with permission from ref 28.

**Figure 7:**
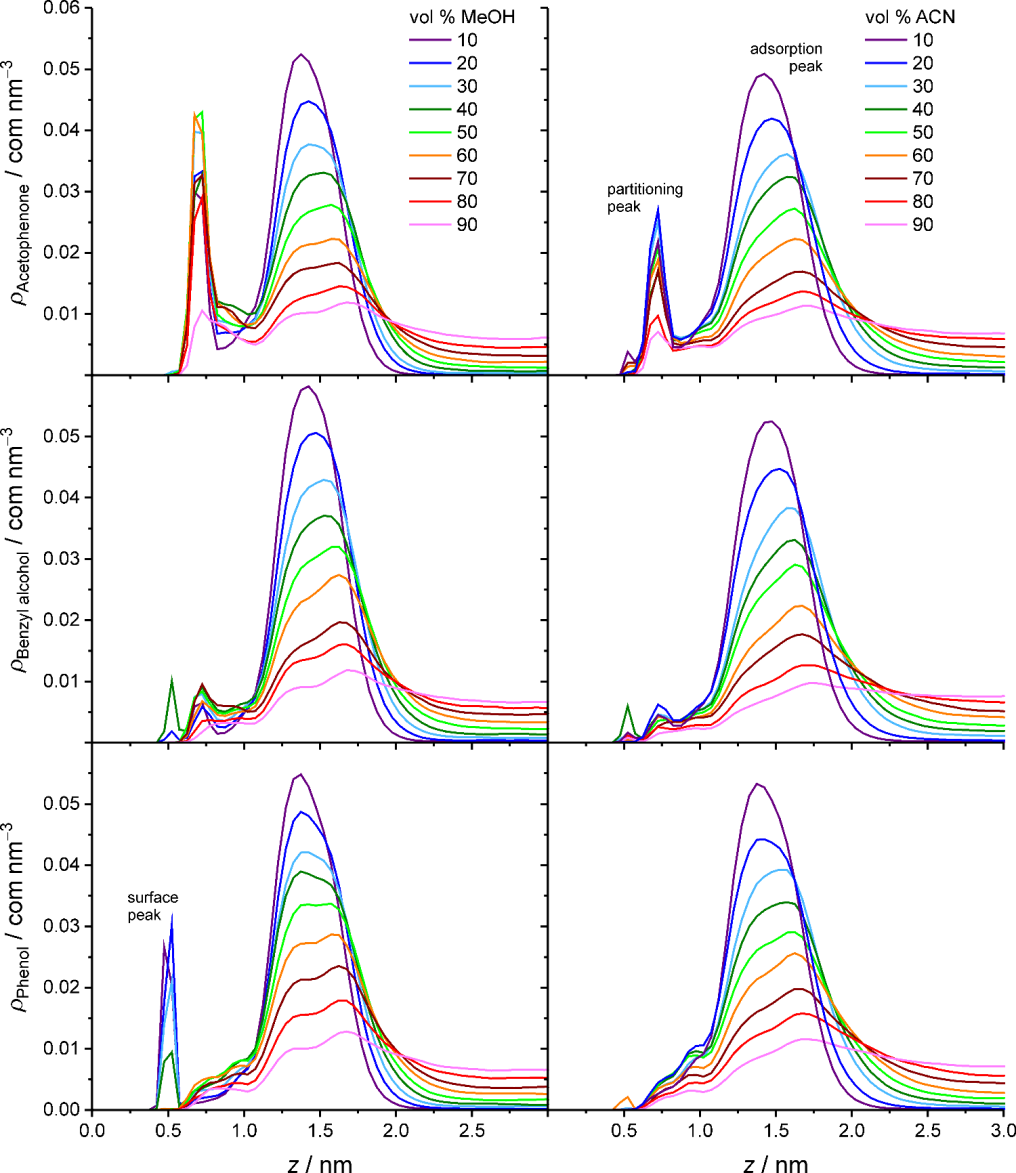
Density profiles for polar analytes in the solvated stationary phase as a function of the mobile-phase parameters.

Figure 7 shows that in the bonded-phase region, acetophenone molecules strongly prefer locations closer to the silica surface (*z* = 0.7 nm), whereas phenol molecules prefer locations closer to the interfacial region (*z* = 0.9 nm), except when the phenol density profile contains a peak at *z* = 0.5 nm that represents solute adsorption to the silica surface. Much smaller surface peaks also appear occasionally in the benzyl alcohol density profiles. In the bonded-phase region, the acetophenone density drops visibly from W–MeOH to W–ACN mobile phases, whereas the phenol and benzyl alcohol density distributions there remain quite unchanged, except for the appearance of phenol surface peaks at highly retentive conditions (i.e., low MeOH volume fractions in the mobile phase).

According to Figures 6 and 7, all compounds of the analyte ensemble prefer solute adsorption to the interfacial region over solute partitioning into the bonded-phase region, but the adsorption preference is much more pronounced for the polar analytes. This analyte density distribution pattern has been consistently observed for small compounds in molecular simulations^17–20,22,24,28,34^ since early studies used the small, flexible solutes *n*-butane and 1-propanol as stand-ins for apolar and polar analytes, respectively.^12–14,16^ The strong preference of polar solutes for the interfacial region has been linked to hydrogen-bond formation with W molecules of the bulk liquid region,^13^ a topic that we examine in the next section. The adsorption of polar solutes to the silica surface was shown to originate from hydrogen-bond formation with residual OH groups.^14^

As opposed to the main retention events in the bonded phase (solute partitioning and solute adsorption), retention events at the silica surface are undesirable as their slow adsorption–desorption kinetics (ms to s range) leads to peak tailing.^44^ Residual OH groups at the silica surface are generally coordinated by solvent molecules so that solute adsorption necessitates the displacement of solvent from the surface-adsorbed layer. As the smallest compound of the polar analytes (Table 1), phenol is the least likely to be hindered by its volume from partial insertion into the surface-adsorbed solvent layer. It is conceivable that the partial charge and associated hydrogen-bond properties of phenol (Table 1) favor surface adsorption. Surface peaks are more likely to appear with W–MeOH mobile phases, when the surface-adsorbed solvent layer contains fewer W molecules than with W–ACN mobile phases (cf. Figure 5 in ref 28). W molecules are much more difficult to displace from the silica surface than MeOH or ACN molecules, which is why in normal-phase chromatography with bare silica columns, where compounds with hydrophilic functional groups are retained by adsorption to the solid silica surface, the OS–OS mobile phase must be free of W traces.^1^

#### 3.2.2 Average Analyte Position in the Solvated Stationary Phase

After pointing out solute-specific differences in the analyte density distributions, we return to a more general point of view. From the first part of the study,^28^ we know that the number of possible bonded-phase contacts for an analyte molecule depends on its distance from the silica surface, *z*_analyte_. A shift of analyte density closer the bulk liquid region, as induced by increasing mobile-phase elution strength, results in a decrease in ⟨*C*_BP_⟩_SP_ and thus a loss of retention. Analogous to ⟨*C*_BP_⟩_SP_, we determined the average analyte position in the solvated stationary phase, ⟨*z*_analyte_⟩_SP_, from the analyte density profiles in Figures 6 and 7. The value of ⟨*z*_analyte_⟩_SP_ quantifies the extent to which the average analyte density in the solvated stationary phase is shifted toward the silica surface and thus indicates the average penetration depth into the bonded phase.

Figure 8 shows the ⟨*z*_analyte_⟩_SP_ values for apolar and polar analytes with W–MeOH and W–ACN mobile phases. The comparison with Table 1 immediately reveals that ⟨*z*_analyte_⟩_SP_ is principally influenced by the HB_solute–W,max_ value of a compound. This means that the hydrogen-bond requirements of an analyte control its average penetration depth into the bonded phase. The comparison of Figure 8 with Figure 1 shows that analyte retention is closely linked to the average penetration depth, as the ⟨*z*_analyte_⟩_SP_ values largely reproduce the retention behavior of the analyte ensemble, with the exception of naphthalene. Lower ⟨*z*_analyte_⟩_SP_ values indicate more analyte density at locations with higher bonded-phase density, which corresponds to higher ⟨*C*_BP_⟩_SP_ values and thus to higher retention factors.

**Figure 8:**
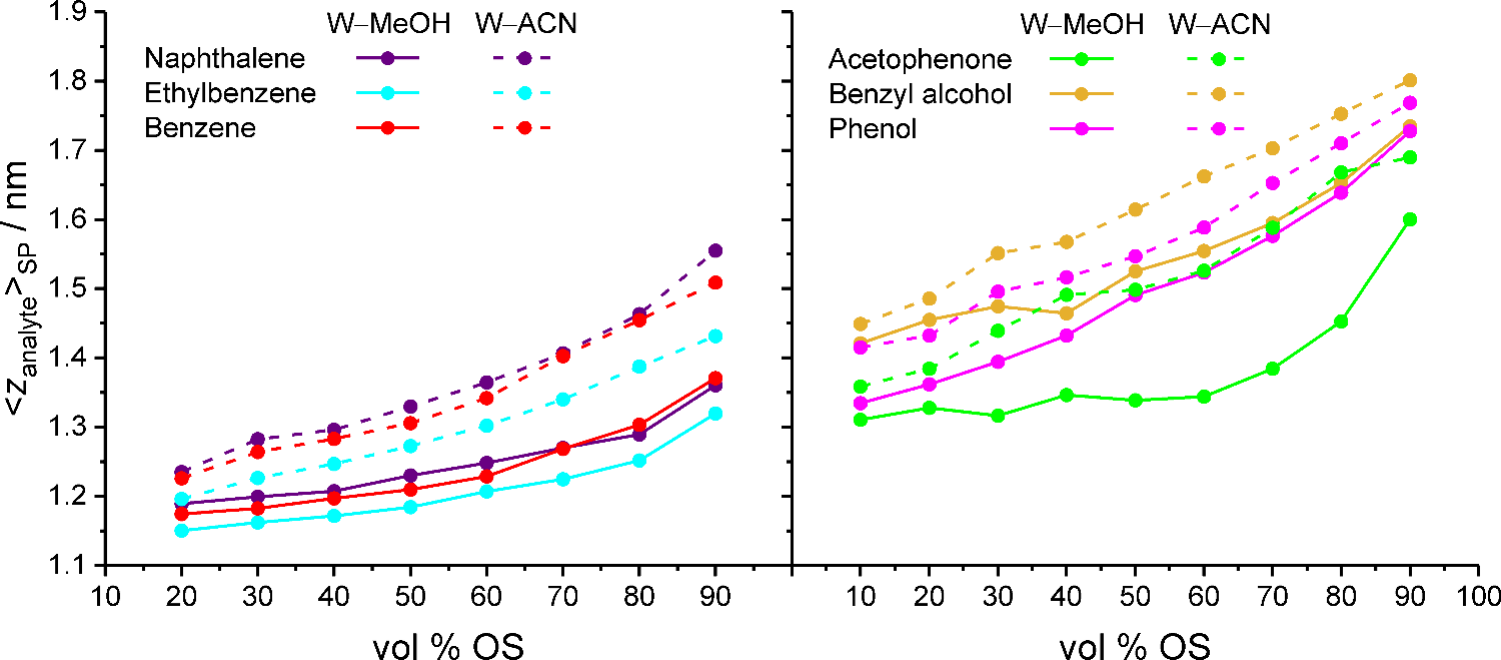
Average position of apolar (left) and polar analytes (right) in the solvated stationary phase, expressed as the average analyte distance from the silica surface, as a function of the mobile-phase parameters.

Corroboration that the average penetration depth into the bonded phase is controlled by the hydrogen-bond requirements of a compound comes from MD simulations of the retention of tryptic peptides from proteomics by a silica-based, C_18_ stationary phase.^45,46^ These peptides are not small molecules but flexible enough to allow partial partitioning into the bonded-phase region. Only segments containing amino acid residues with hydrophobic side chains partition into the bonded phase, while peptide segments containing hydrophilic side chains (and charges) that require hydrogen-bond coordination are kept at the edge of the interfacial region.

Because the majority of analyte density is located in the adsorption peak (Figures 6 and 7), the value of ⟨*z*_analyte_⟩_SP_ is primarily determined by the contribution from the adsorption peak in the interfacial region to the analyte density in the solvated stationary phase, shown in Figure 9. The idiosyncratic evolution of the adsorption contribution curves for acetophenone and benzyl alcohol with W–MeOH mobile phases explains why the corresponding ⟨*z*_analyte_⟩_SP_ curves do not follow the otherwise observed, straightforward increase with an increasing OS volume fraction in the mobile phase. Secondly, the value of ⟨*z*_analyte_⟩_SP_ is influenced by how analyte density is distributed within the interfacial region. Because naphthalene has more density in the silica-surface side of the adsorption peak than benzene (Figure 6), the ⟨*z*_analyte_⟩_SP_ values of benzene can approach or overtake those of naphthalene at high OS volume fractions in the mobile phase, although naphthalene has higher hydrogen-bond requirements and a larger adsorption contribution than benzene.

**Figure 9:**
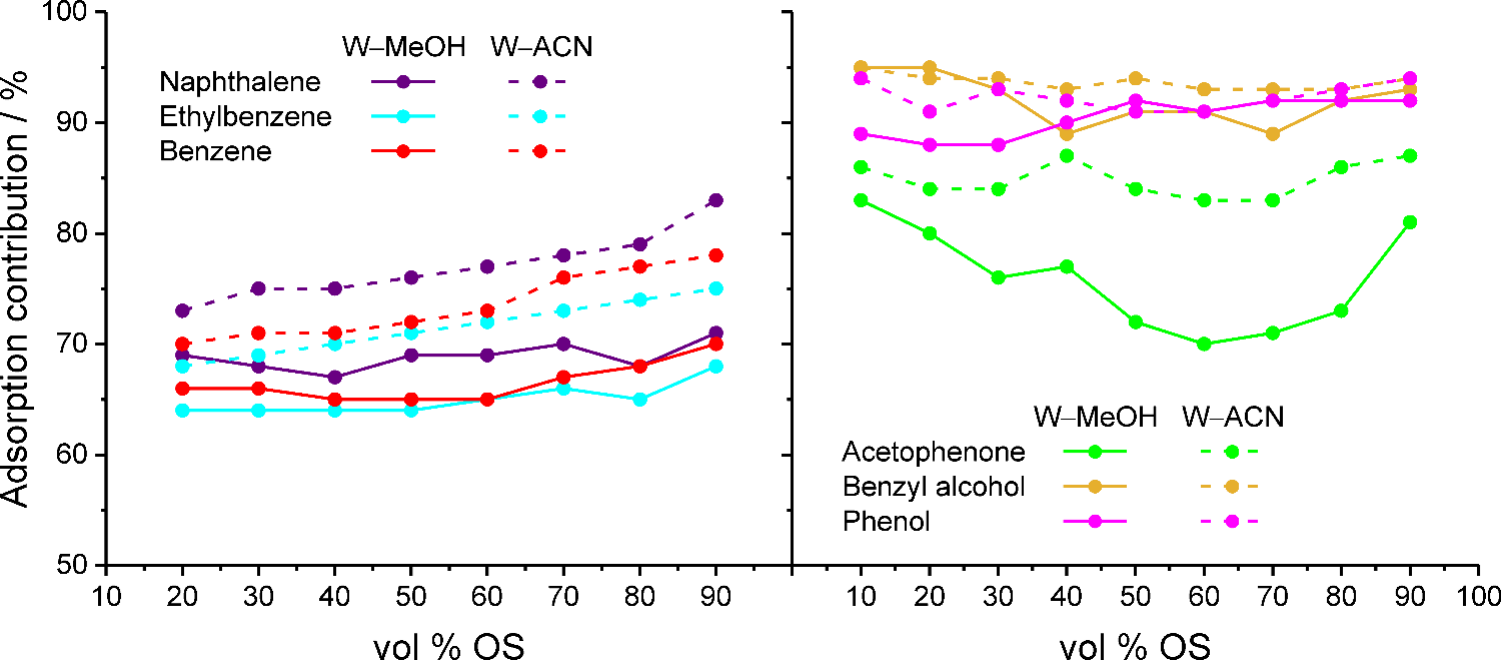
Contribution from the adsorption peak in the interfacial region to the density of apolar (left) and polar analytes (right) in the solvated stationary phase as a function of the mobile-phase parameters.

Figure 9 shows that the adsorption contributions of the apolar analytes increase with the OS eluent strength in the mobile phase and also from 20 to 90 vol % OS (though not strictly in-between with W–MeOH mobile phases), whereas the adsorption contributions of the polar analytes do not share a uniform response to the mobile-phase parameters. Acetophenone responds more strongly to the OS type in the mobile phase than any other compound in the ensemble, as already evidenced in Figure 7 by the dramatic loss of the partitioning peak density from W–MeOH to W–ACN mobile phases. Averaged over the vol % OS range, the adsorption peak of acetophenone contributes 75 and 85% of the analyte density in the solvated stationary phase with W–MeOH and W–ACN mobile phases, respectively. Regarding the analyte density distribution between bonded-phase and interfacial regions, acetophenone is therefore closer to the apolar analytes with W–MeOH mobile phases (e.g., naphthalene with an average adsorption contribution of 68%) and closer to the polar analytes with W–ACN mobile phases. In contrast, the average adsorption contributions of phenol and benzyl alcohol are barely affected by the OS type and remain at 90 and 92%, respectively, with W–MeOH mobile phases and at 92 and 93% with W–ACN mobile phases. A distinctive feature of the adsorption contribution curves for phenol and benzyl alcohol is their divergence at low MeOH volume fractions in the mobile phase, which mirrors the onset of *k*(benzyl alcohol) > *k*(phenol) in the experimental retention data (cf. Figure 1 and Table S1). We will return to this observation below.

### 3.3 Solute–Solvent Hydrogen Bonding in the Solvated Stationary Phase

The solute-specific differences between the analyte density distributions strongly point to solute–solvent hydrogen bonding as the main contributor to differential analyte retention. This topic has previously been discussed as a feature that distinguishes the retention behavior of apolar vs polar solutes.^12–20^ In this section, we will show that (i) solute–solvent hydrogen bonding in the solvated stationary phase influences the density distribution of every compound in the analyte ensemble and (ii) the retention behavior of the polar analytes depends critically on the specific hydrogen-bond requirements of a compound.

#### 3.3.1 Average Number of Solute–Solvent Hydrogen Bonds in the Solvated Stationary Phase

Complementary to the stationary phase-averaged values for bonded-phase contacts and analyte positions in Figures 4 and 8, respectively, Figure 10 displays the stationary phase-averaged number of solute–solvent hydrogen bonds per analyte molecule, ⟨HB_solute–solvent_⟩_SP_, as well as the fraction of solute–solvent hydrogen bonds in the bulk liquid region, HB_solute–solvent,bulk_, recovered by analyte molecules in the solvated stationary phase. Values for HB_solute–solvent,bulk_ were available from our preceding MD simulation study of the solute solvation shells.^2^

**Figure 10:**
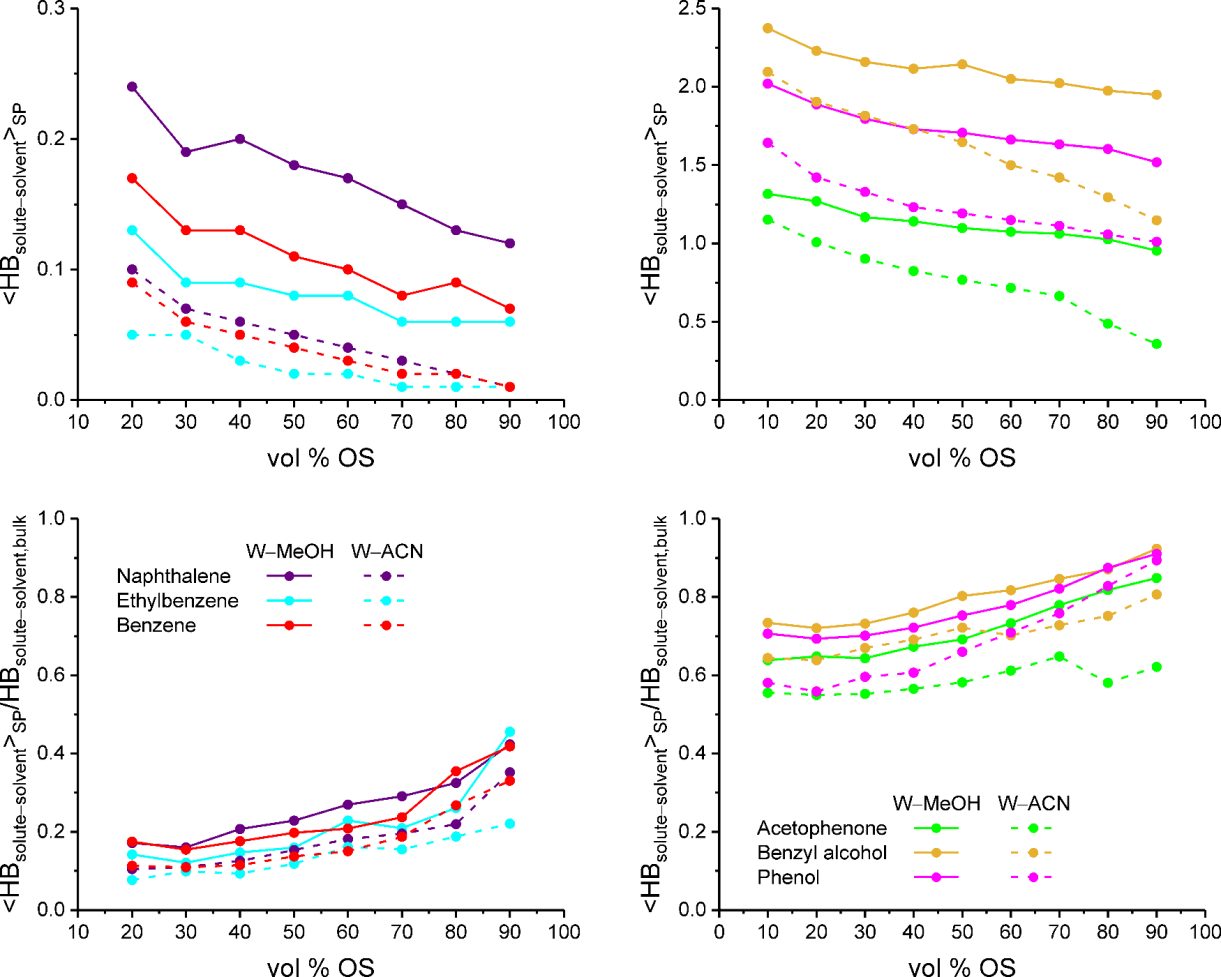
Average number of solute–solvent hydrogen bonds per analyte molecule for apolar (left) and polar analytes (right) in the solvated stationary phase (top) and fraction of bulk liquid solute–solvent hydrogen bonds per analyte molecule recovered in the solvated stationary phase (bottom) as a function of the mobile-phase parameters.

The order of the ⟨HB_solute–solvent_⟩_SP_ values in Figure 10 reflects the respective HB_solute–solvent,max_ values of the compounds (Table 1), confirming that solute-specific differences regarding the extent of solute–solvent hydrogen bonding displayed in neat W are largely maintained in the stationary phase. The only deviation from this rule is observed for benzene, which has larger ⟨HB_solute–solvent_⟩_SP_ values than ethylbenzene at equal HB_solute–solvent,max_ values. This is explained by the higher adsorption contribution of benzene compared with that of ethylbenzene (Figure 9). As most of the solvent density in the stationary phase is located in the interfacial region, benzene can recover a larger fraction of its bulk liquid solute–solvent hydrogen bonds than ethylbenzene.

Figure 10 also shows that the distinction between apolar and polar analytes regarding the extent of solute–solvent hydrogen bonding is considerably sharper in the solvated stationary phase than in the bulk liquid region. The apolar analytes maintain a very low level of hydrogen bonding in the stationary phase, as shown by the absolute ⟨HB_solute–solvent_⟩_SP_ values as well as the bulk liquid recovery fractions. In contrast, the polar analytes recover typically more than 70%, and at least more than 50%, of their HB_solute–solvent,bulk_ values in the stationary phase, depending on the OS type in the mobile phase.

As expected, the value of ⟨HB_solute–solvent_⟩_SP_ generally decreases from W–MeOH to W–ACN mobile phases, that is, with the total solvent density in the solvated stationary phase (cf. Figures 4 and 5 in ref 28). Additionally, the exchange of MeOH for ACN as the OS limits the options for analyte compounds that require donor solvents for hydrogen-bond formation. In Figure 10, this is particularly obvious for acetophenone, whose ⟨HB_solute–solvent_⟩_SP_ values drop at >70 vol % ACN, but not at >70 vol % MeOH. The lack of sufficient access to donor solvents in the bonded-phase region, when the stationary phase is equilibrated with W–ACN mobile phases, explains the visibly reduced partitioning peak in the acetophenone density profiles (Figure 7).

Another detail in Figure 10 related to the mobile-phase sensitivity of the polar analytes is the shallow decline of the ⟨HB_solute–solvent_⟩_SP_ curve for phenol at >50 vol % ACN compared with the steeper decline of the ⟨HB_solute–solvent_⟩_SP_ curve for benzyl alcohol. The behavior is reflected by the recovery curves for the two compounds, where the recovery values of phenol overtake those of benzyl alcohol at >60 vol % ACN. This indicates that the hydrogen-bond coordination of phenol molecules in the solvated stationary phase is less affected than that of benzyl alcohol molecules when the supply of donor solvents becomes scarce, in agreement with the respective hydrogen-bond requirements of the two compounds (Table 1).

Overall, Figure 10 proves that analyte molecules in the solvated stationary phase maintain the solute-specific hydrogen bonding patterns observed in the bulk liquid region, whereby hydrogen bonds involving functional groups XH*_n_* are recovered to a larger extent than hydrogen bonds involving π electrons. The presence and hydrogen-bond requirements of a functional group in a compound thus considerably influence its analyte density distribution in the solvated stationary phase and ultimately its retention factor.

#### 3.3.2 Hydrogen-Bond Partner Density Profiles

After having established that the hydrogen-bond coordination of functional groups is of high consequence to the retention of polar analytes, we take a closer look at how hydrogen bonding opportunities, or lack thereof, shape the density profiles for the polar analytes (Figure 7). Figure 11 illuminates the solute-specific differences between the polar analytes from the density distributions of their hydrogen-bond partners, shown separately for the partitioning peak and the adsorption peak, at 50 vol % OS in the mobile phase. The hydrogen-bond partner density profiles are annotated with snapshots that visualize the preferential analyte orientation at a given location. The associated probability distributions for the angle between the molecular vector of an analyte molecule and the surface normal are shown in Figures S1 and S2 in the Supporting Information.

**Figure 11:**
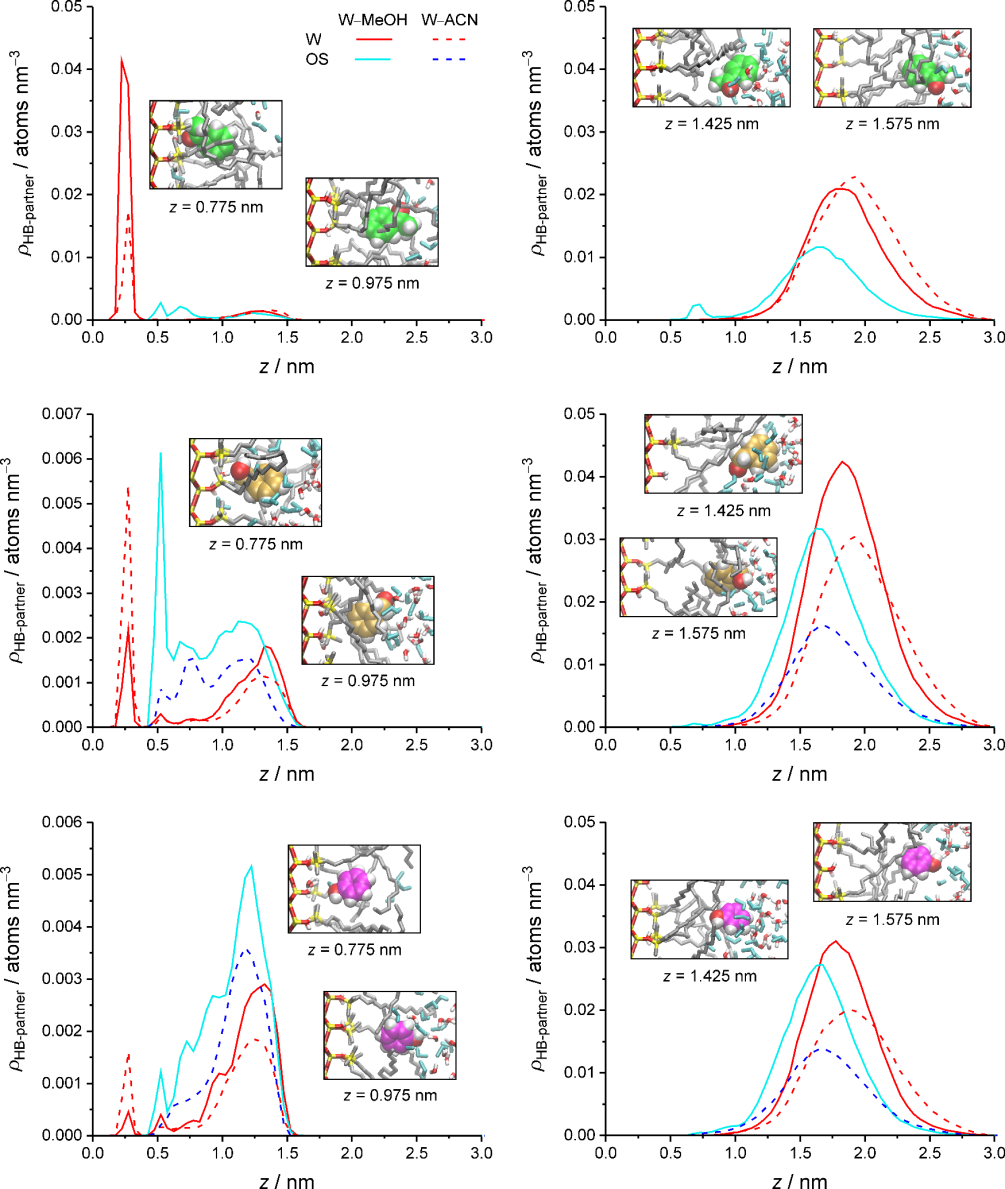
Density distribution of the hydrogen-bond partners for polar analyte molecules (from top to bottom: acetophenone, benzyl alcohol, and phenol) in the partitioning peak (left) and the adsorption peak (right) at 50 vol % OS in the W–MeOH (solid lines) or W–ACN mobile phase (dashed lines). Snapshots from the simulations with the W–MeOH mobile phase visualize the preferential analyte orientation at the indicated positions.

Before we discuss Figure 11, we briefly recall the average solvent densities in the bonded-phase and interfacial region as established in the first part of the study.^28^ W density dominates the surface-adsorbed solvent layer up to 80 vol % MeOH and >90 vol % ACN in the mobile phase but is otherwise scarce in the bonded-phase region. In the interfacial region, the W density drops below the OS density at 60 vol % MeOH and ∼55 vol % ACN in the mobile phase. The average W and OS densities in the interfacial region are approximately 9.2 and 6.8 atoms nm^–3^, respectively, at 50 vol % MeOH, and 7.1 and 5.8 atoms nm^–3^ at 50 vol % ACN in the mobile phase (cf. Figure 5 in ref 28).

Figure 11 shows that polar analyte molecules in the partitioning peak find hydrogen-bond partners from the surface-adsorbed solvent layer up to *z* = 1.6 nm in the interfacial region, whereby the preferential analyte orientation is arranged to facilitate hydrogen-bond formation with the preferred partner molecules. Acetophenone molecules in the partitioning peak point the functional group predominantly to the silica surface because they rely overwhelmingly on surface-adsorbed W molecules as hydrogen-bond partners. A second, smaller fraction of acetophenone molecules forms hydrogen bonds with MeOH molecules in the solvent-depleted bonded-phase region (at *z* = 0.55 and 0.7 nm). A third, small fraction of acetophenone molecules turns the functional group toward the bulk liquid region because their location allows them to reach solvent molecules of the interfacial region (*z* = 1.05–1.6 nm).

Phenol and benzyl alcohol molecules in the partitioning peak rely much less on surface-adsorbed W molecules as hydrogen-bond partners than acetophenone molecules and accept a larger OS contribution to their hydrogen-bond coordination. Benzyl alcohol molecules are approximately as likely to turn their functional group toward the silica surface as in the opposite direction. Their hydrogen-bond partners are surface-adsorbed W molecules or OS molecules of the adjacent layers (at *z* = 0.5 and 0.75 nm) in the first case and solvent molecules of the interfacial region in the second case. Phenol molecules in the partitioning peak prefer to direct their functional group away from the silica surface for hydrogen-bond formation with solvent molecules, predominantly OS molecules, in the interfacial region.

The hydrogen-bond partner density profiles for the partitioning peak closely reflect the solute-specific differences between the analyte density distributions in the bonded-phase region (Figure 7). In contrast, the hydrogen-bond partner density profiles for the adsorption peak, which holds the majority of the analyte density, are quite similar for the three compounds. The majority of hydrogen-bond partners in the adsorption peak is found within *z* = 1.5–2.25 nm. Polar analyte molecules located at *z* > 1.5 nm predominantly point the functional group to the bulk liquid region and thus in the direction of increasing W density. A smaller fraction of polar analyte molecules, located at *z* < 1.5 nm, forms hydrogen bonds with W and OS molecules in the inner part of the interfacial region. A very small fraction of analyte molecules forms hydrogen bonds with OS molecules in the bonded-phase region; this is best visible in the hydrogen-bond partner density profile for acetophenone. Although the hydrogen-bond partner density profiles extend into the bulk liquid region (*z* > 2.55 nm), polar analyte molecules find their hydrogen-bond partners mainly within the interfacial region.

The observation that the bulk liquid region contributes comparatively little to the hydrogen-bond coordination of polar analyte molecules in the adsorption peak underlines the importance of the stationary-phase solvation for the retention of polar analytes in RPLC. If hydrogen-bond partners were found only at the silica surface or in the bulk liquid region, polar analytes would have much less density in the stationary phase and thus be much less retained than they are on C_18_ columns. The ensuing retention loss would severely narrow the RPLC application spectrum in practice, given that solutes with hydrophilic functional groups abound in samples from drug discovery, environmental monitoring, and food chemistry.^47–49^ The importance of the stationary-phase solvation for the retention of polar analytes also explains why their elution order is typically better predicted by the log *K*_OW_ values than by the log *K*_HW_ values of the solutes. When equilibrated with W, *n*-octanol takes up considerable W density,^50^ which has been shown to favor the partitioning of polar solutes into the “wet” *n*-octanol chains,^51^ whereas W molecules barely enter the *n*-hexadecane chains.^52^ Another confirmation is the increased retention of polar analytes on polar-embedded vs C_18_ stationary phases, which originates from the W density associated with the polar functional group embedded into the alkyl chain, as revealed by molecular simulations.^53^

The comparison of Figures 11 and 7 demonstrates that the hydrogen-bond partner density profiles closely reflect the respective analyte density profiles. Higher analyte density at a given location in the solvated stationary phase is generally accompanied by higher hydrogen-bond partner density, indicating that the local density of polar analyte molecules is limited by the availability of suitable hydrogen-bond partners. The dramatic loss of partitioning peak density observed for acetophenone from W–MeOH to W–ACN mobile phases (Figure 7) is thus owing to the concurrent loss of hydrogen-bond donor density in the bonded-phase region. However, even with W–MeOH mobile phases, acetophenone relies much more on solute–W hydrogen bonds than phenol and benzyl alcohol.

Figure 12 compares the polar analytes regarding the W contributions to ⟨HB_solute–solvent_⟩_SP_ and HB_solute–solvent,bulk_ over the whole vol % OS range. Except when W is the only possible hydrogen-bond partner, such as for acetophenone with W–ACN mobile phases, the W contribution to ⟨HB_solute–solvent_⟩_SP_ is lower than the W contribution to HB_solute–solvent,bulk_, which shows that analyte molecules adjust their hydrogen-bond partner preference to the environment. The extent of this adjustment is, however, solute-specific. Acetophenone shows the highest W contributions of the three compounds and maintains a preference for solute–W hydrogen bonds beyond the point where W molecules outnumber MeOH molecules in the solvated stationary phase or the bulk liquid region (at ∼60 and 70 vol % MeOH in the mobile phase, respectively). The pronounced preference of acetophenone for solute–W hydrogen bonds is not solely due to the compound’s requirements toward donor solvents but reflects that W molecules are better hydrogen-bond donors than MeOH molecules for the carbonyl group.^2^ The difference between phenol and benzyl alcohol regarding the W contribution to ⟨HB_solute–solvent_⟩_SP_, on the other hand, reflects that, due to its higher requirements toward donor solvents, benzyl alcohol has less scope than phenol for adjusting its hydrogen-bond partner preference in the solvated stationary phase. In the bulk liquid region, where access to W molecules is not determined by the bonded-phase density, phenol and benzyl alcohol share the same hydrogen-bond partner preferences.

**Figure 12:**
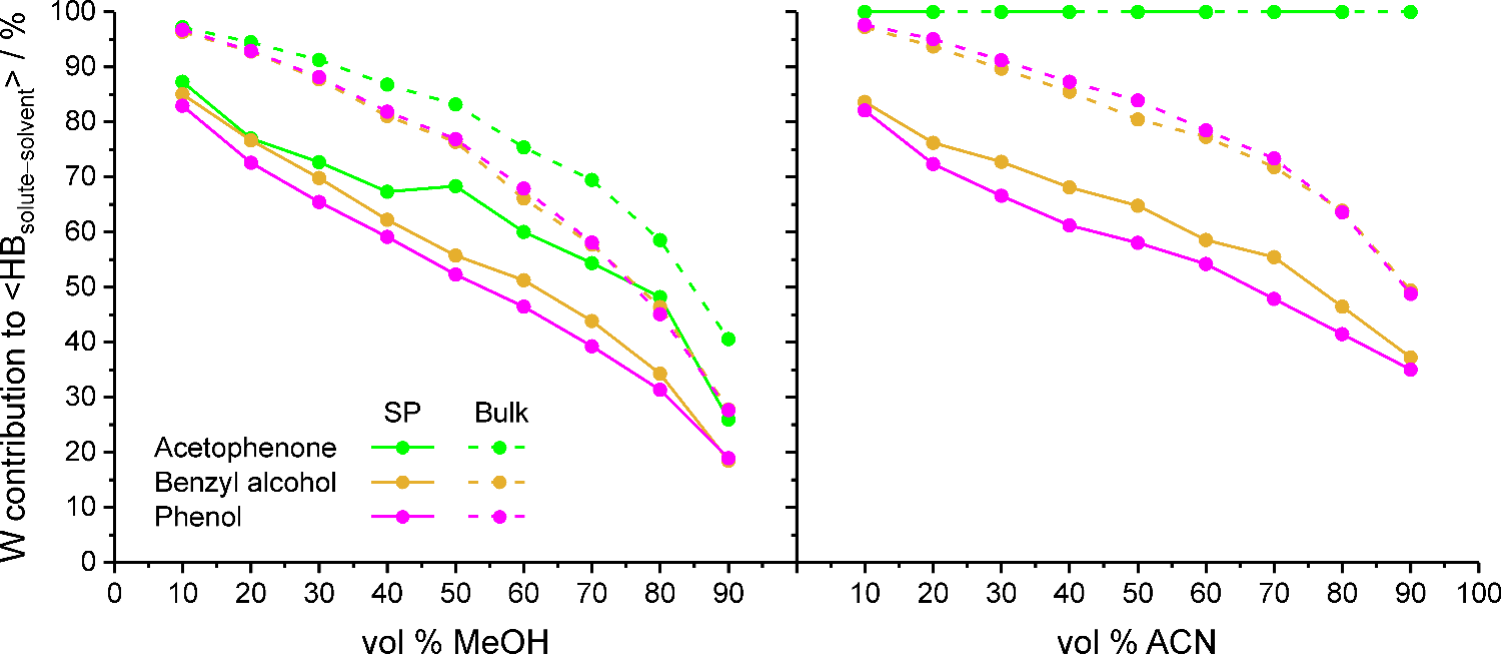
Contribution of W molecules to the average number of solute–solvent hydrogen bonds per analyte molecule for polar analytes in the solvated stationary phase (solid lines) and the bulk liquid region (dashed lines) as a function of the mobile-phase parameters.

Overall, the analysis of solute–solvent hydrogen bonding has shown that the density of polar analytes in the solvated stationary phase is limited by the density of suitable hydrogen-bond partners, particularly the W density. Because the solvent density distribution in the solvated stationary phase is a function of the mobile-phase elution strength, as shown in the first part of the study,^28^ the differential retention behavior of polar analytes is sensitive to the OS volume fraction and OS type in the mobile phase.

### 3.4 Analyte Selectivity

So far, the results of this study were presented to emphasize the generalities of solute-specific RPLC retention. We identified two major influences on the analyte density distribution, which both depend on the stationary-phase solvation and thus the mobile-phase elution strength: evasion of W contacts by the hydrophobic structural elements and the importance of solute–solvent hydrogen bonds for the hydrophilic structural elements of analyte molecules. Higher W density in the solvated stationary phase, associated with lower mobile-phase elution strength, drives analyte molecules deeper into the bonded phase to avoid W contacts and at the same time raises the extent to which the hydrogen-bond requirements of analyte molecules are met. That is why a lower mobile-phase elution strength increases the stationary phase-averaged number of bonded-phase contacts per analyte molecule and thus the retention factor in general. We now apply this knowledge to explain the retention behavior of the analyte ensemble shown in Figures 1 and 4.

#### 3.4.1 Differential Retention of Apolar Analytes

As mentioned in the Introduction, the separation of alkylbenzene homologues is a performance test for RPLC columns in practice.^9^ In our study, the comparison of benzene and ethylbenzene retention exemplifies how the column discriminates between structurally highly similar, apolar compounds. Ethylbenzene has more hydrophobic structural elements, *N*(CH*_*x*_*), than benzene at an equal HB_solute–W,max_ value (Table 1). According to the ⟨*z*_analyte_⟩_SP_ values shown in Figure 8, ethylbenzene density is shifted closer toward the silica surface, and thus toward environments of higher bonded-phase density, than benzene density. This shows that a higher number of hydrophobic structural elements at equal hydrogen-bond requirements shifts analyte density deeper into the bonded phase, away from the W density in the interfacial region. This principle is further corroborated by the preferential orientation of the ethylbenzene molecules in the adsorption peak. The ethyl side chain is directed toward the silica surface and thus away from the influx of W molecules into the interfacial region. In contrast, benzene and naphthalene molecules, which have no side chains, show no preferential orientation at any location in the stationary phase (probability distributions for the angle between the molecular vector and the surface normal are shown in Figures S3 and S4).

Additionally, a higher number of hydrophobic structural elements translates to a higher number of bonded-phase contacts at a given location in the solvated stationary phase. Alkyl groups that increase the bonded-phase affinity of a solute without altering its hydrogen-bond requirements have therefore a 2-fold effect on the average number of bonded-phase contacts, which explains the high methylene selectivity observed in RPLC separations of alkylbenzenes.

The coelution of ethylbenzene and naphthalene is an undesired consequence of the interplay between bonded-phase affinity and hydrogen-bond requirements. Due to the larger hydrogen-bond requirements of naphthalene (Table 1), naphthalene density is shifted further from the silica surface than ethylbenzene density, toward environments of lower bonded-phase density, but the higher bonded-phase affinity of naphthalene almost compensates for this density shift, resulting in similar ⟨*C*_BP_⟩_SP_ values for ethylbenzene and naphthalene (cf. Figure 4).

The analyte density distributions and ⟨*z*_analyte_⟩_SP_ values of ethylbenzene and naphthalene (Figures 6 and 8) do not show enough sensitivity to the mobile-phase elution strength to propose a selectivity improvement through the mobile-phase parameters, necessitating a change of the stationary-phase chemistry. In RPLC practice, the analyte pair ethylbenzene/naphthalene and its analog, butylbenzene/anthracene, are separated on phenyl columns, which are assumed to discriminate between compounds based on π–π interactions.^54,55^

#### 3.4.2 Differential Retention of Polar Analytes

Because the polar analytes differ not only in their *N*(CH*_*x*_*) and HB_solute–solvent,max_ values but also their hydrogen-bond partner preferences, as discussed in Section 3.3.2, their retention behavior cannot be fully explained by the derived rule that analyte retention increases with the bonded-phase affinity and decreases with the hydrogen-bond requirements of a solute. The dependence on suitable hydrogen-bond partners, however, enables the manipulation of analyte selectivity through the mobile-phase parameters.

Acetophenone has a higher bonded-phase affinity and lower hydrogen-bond requirements than benzyl alcohol and phenol and is accordingly more retained on the C_18_ column. However, due to the dependence from donor solvents, the acetophenone density distribution is highly sensitive to the hydrogen-bond properties of the OS in the mobile phase, which has a large impact on the diffusive mass transport of acetophenone molecules through the mesopore space of a column. Multiscale simulations of diffusive transport in the reconstructed pore space of monolithic silica columns have recently shown that the effective mesopore diffusivities of the apolar analytes in the ensemble are below those of the polar analytes because lower partitioning peak densities favor the mesopore diffusivity.^27^ The effective mesopore diffusivities of acetophenone were below those of phenol with W–MeOH mobile phases but comparable to those of phenol with W–ACN mobile phases. We now know that the loss of partitioning peak density leading to higher mesopore diffusivity is caused by the lack of suitable hydrogen-bond partners for acetophenone molecules in the bonded-phase region.

Although the adsorption contributions of phenol and benzyl alcohol are rather insensitive to the OS type in the mobile phase, the differential retention of these two compounds hinges on the density of hydrogen-bond donor solvents. Compared with phenol, benzyl alcohol requires more solvent molecules, specifically hydrogen-bond donor molecules, for coordination of its functional group but has an additional methylene group and thus higher bonded-phase affinity (Table 1). The mobile-phase parameters determine the extent to which the hydrogen-bond requirements of a solute are met and thus decide about the extent to which analyte density in the solvated stationary phase is limited.

With W–ACN mobile phases, the hydrogen-bond donor density in the solvated stationary phase is much lower than that with W–MeOH mobile phases. W–ACN mobile phases offer no hydrogen-bond donor possibilities by the OS and generate less W density in the solvated stationary phase than W–MeOH mobile phases, particularly in the crucial interfacial region (cf. Figures 4 and 5 in ref 28), where phenol and benzyl alcohol have >90% of their analyte density. Due to the higher requirements of benzyl alcohol toward donor solvents, benzyl alcohol density in the solvated stationary phase is more limited than phenol density, so that benzyl alcohol is less retained than phenol with W–ACN mobile phases (Figure 1).

The density limitation is alleviated by the higher hydrogen-bond donor density generated by W–MeOH mobile phases in the solvated stationary phase. With mobile phases containing >20 vol % MeOH, phenol and benzyl alcohol coelute from the C_18_ column because the higher bonded-phase affinity of benzyl alcohol is now counterbalanced by the lower hydrogen-bond requirements of phenol. Only at low MeOH volume fractions in the mobile phase and thus high W density in the solvated stationary phase is the density limitation for benzyl alcohol fully lifted, so that benzyl alcohol becomes more retained than phenol on the C_18_ column, and the methylene selectivity for this analyte pair is restored. The density evolution of benzyl alcohol vs phenol is reflected in the diverging adsorption contribution curves for the two compounds toward low MeOH volume fractions (Figure 9). The rise in the adsorption contribution of benzyl alcohol at low MeOH volume fractions in the mobile phase originates from increased analyte density in the adsorption peak, enabled by increased W density in the interfacial region associated with lower mobile-phase elution strength. The fact that low OS volume fractions in the mobile phase are required to fully lift the benzyl alcohol density limitation, even when the OS has donor properties, emphasizes that the W density in the solvated stationary phase is the critical system property to satisfy the hydrogen-bond requirements of polar analytes.

The critical importance of the W density in the solvated stationary phase for the separation of the analyte pair phenol/benzyl alcohol is corroborated by Figure 13, which shows the evolution of the hydrogen-bond partner densities of phenol and benzyl alcohol molecules in the adsorption peak with increasing OS volume fraction in the mobile phase. The data were generated by the integration of peak areas in the respective hydrogen-bond partner density profiles (Figures S5 and S6 in the Supporting Information). Figure 13 confirms that the two compounds differ much more in their W hydrogen-bond partner densities than in their OS hydrogen-bond partner densities. The point where benzyl alcohol is more retained than phenol is particularly well-reflected by the surge of W hydrogen-bond partner density at <20 vol % MeOH. The mobile-phase sensitivity of the analyte pair phenol/benzyl alcohol originates therefore from the extent to which OS molecules drag W molecules into the stationary phase rather than from the OS contribution to the hydrogen-bond coordination of the solutes and is thus related to W–OS rather than solute–OS hydrogen bonding.

**Figure 13:**
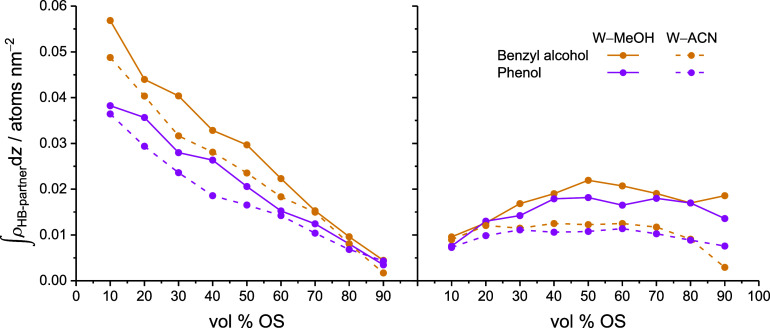
W and OS hydrogen-bond partner density (left and right, respectively) for phenol and benzyl alcohol molecules in the adsorption peak as a function of the mobile-phase parameters.

## 4 Conclusions

In this study, we investigated the mobile-phase contribution to differential analyte retention in RPLC through MD simulations performed for an analyte ensemble in a well-established slit-pore model of a silica-based, endcapped, C_18_ stationary phase equilibrated with W–MeOH and W–ACN mobile phases over a wide range of solvent compositions. The parameters of the study were informed by the conditions under which small, neutral compounds of low to moderate solute polarity are typically separated in HPLC practice. The results challenge existing ideas about the mechanism of RPLC retention, the role of the mobile phase in RPLC separations, and the solute polarity concept, while at the same time confirming assumptions about the nature of RPLC retention.

From a fundamental point of view, the most important result of this study is the recovery of the experimentally observed retention behavior by the stationary phase-averaged number of bonded-phase contacts per analyte molecule. This reflects that the nature of RPLC retention is defined by interactions between analyte molecules and hydrophobic bonded-phase groups, confirming a long-held belief in the chromatographic community.^1^ The stationary phase-averaged number of bonded-phase contacts is the molecular-level equivalent of the retention factor, but whereas the latter simply quantifies how much of the analyte density is located in the stationary-phase compartment of the column, the former also quantifies how the analyte density is distributed within the stationary-phase compartment. The bonded-phase density decreases from the bonded-phase region over the interfacial region to zero in the bulk liquid region. Therefore, the number of bonded-phase contacts for a given compound increases with its average penetration depth into the bonded-phase chains.

The governing principle behind differential analyte retention is the solute-specific response to the W density distribution in the system, which is a function of the OS volume fraction and the OS type in the mobile phase. Analyte molecules retreat into the hydrophobic bonded phase to protect their hydrophobic structural elements from contact with W molecules but at the same time have to maintain W contacts to satisfy their hydrogen-bond requirements. Through the W density distribution in the solvated stationary phase, the mobile phase controls the average penetration depth of a compound into the bonded phase, which, in turn, informs the number of bonded-phase contacts per analyte molecule and thus the analyte retention factor. Compounds with a higher number of hydrophobic structural elements make more bonded-phase contacts at a given location in the solvated stationary phase. This is why compounds that differ in molecular structure have solute-specific analyte density distributions but can still coelute.

Although RPLC retention is defined by analyte–bonded-phase contacts, the hydrogen-bond requirements of a compound are key to its RPLC retention behavior because the analyte density in the solvated stationary phase is limited by the density of suitable hydrogen-bond partners. This opens a route to manipulating the selectivity via the mobile-phase parameters that control stationary-phase solvation. Changing from MeOH to ACN as OS decreases the hydrogen-bond donor density in the solvated stationary phase, which discriminates against compounds with higher requirements toward donor solvents.

In summary, our study has shown that the mobile phase influences the solvent, bonded-phase, and analyte density at every location in the system, controls analyte retention, and modifies the selectivity. Importantly, analyte retention and selectivity are impacted via the solvation of the stationary phase rather than the bulk liquid solvation of the analytes. The revelation of the mobile phase as the dynamic component of RPLC separations has important implications for column selection and retention prediction. First, the hydrogen-bond properties of the compounds should be considered in addition to the log *K*_OW_ values to overcome the limitations of the solute polarity concept. Second, the mobile-phase composition should be selected in view of stationary-phase solvation. Third, more effort should be directed into establishing the solvation of different stationary-phase chemistries available for RPLC separations.

## Supplementary Material



## Data Availability

Input files for the MD simulations of this study are openly available in the Zenodo repository via the DOI: 10.5281/zenodo.15364716 under the license CC-BY-4.0 (Creative Commons Attribution 4.0 International).

## Notes

Selectivity factors for the critical analyte pairs (Table S1); location of the stationary-phase limit (Tables S2 and S3); cutoff radii for solute–solvent hydrogen bonds (Table S4); orientation of polar analyte molecules in the partitioning and adsorption peaks (Figures S1 and S2); orientation of apolar analyte molecules in the partitioning and adsorption peaks (Figures S3 and S4); W and OS hydrogen-bond partner density profiles for polar analyte molecules in the adsorption peak (Figures S5 and S6) (PDF)

## Abbreviations

ACN: acetonitrile
BP: bonded phase
C_18_: dimethyl-*n*-octadecylsilane
com: center-of-mass
HB: hydrogen-bond
HILIC: hydrophilic interaction liquid chromatography
HPLC: high-performance liquid chromatography
MeOH: methanol
MD: molecular dynamics
MP: mobile phase
OS: organic solvent
RDF: radial distribution function
RPLC: reversed-phase liquid chromatography
SP: stationary phase
W: water
