# Supplementary material for: Mobile-Phase Contributions to Analyte Retention and Selectivity in Reversed-Phase Liquid Chromatography: 2. Solute-Specific Effects
Source: J Phys Chem B. 2025 Jun 16;129(25):6401–18. doi: 10.1021/acs.jpcb.5c01697 (PMC12207576; doi:10.1021/acs.jpcb.5c01697)

Supporting Information

Mobile-Phase Contributions to Analyte Retention and Selectivity in Reversed-Phase Liquid Chromatography: 2. Solute-Specific Effects

Andreas Steinhoff, Alexandra Höltzel, and Ulrich Tallarek *

Department of Chemistry, Philipps-Universität Marburg, Hans-Meerwein-Strasse 4, 35032 Marburg, Germany

* Email: tallarek@staff.uni-marburg.de

Table S1. Selectivity factors α for the analyte pairs ethylbenzene/naphthalene and phenol/benzyl alcohol with W–MeOH and W–ACN mobile phases.

	α(Ethylbenzene/Naphthalene) [a]		α(Phenol/Benzyl alcohol) [a]	
Vol % OS	W–MeOH	W–ACN	W–MeOH	W–ACN
10	–	–	1.24 [c]	1.23
20	–	–	1.08 [c]	1.51
30	–	1.01	1.02 [c]	1.58
40	1.10 [b]	1.11	1.01	1.50
50	1.01	1.05	1.01	1.46
60	1.07	1.06	1.00	1.35
70	1.09	1.05	1.02	1.26
80	1.09	1.06	1.04	1.13
90	1.07	1.04	1.00	1.00

[a] Selectivity factors α were calculated from the experimental retention factors k of the compounds as shown in Figure 1 via eq 3 in the main text. [b] k(naphthalene) > k(ethylbenzene). [c] k(benzyl alcohol) > k(phenol).

Table S2. Location of the stationary-phase limit, z_{SP}, for apolar analytes at a given OS volume fraction in the W–MeOH or W–ACN mobile phase.

Vol % OS	Naphthalene	Ethylbenzene	Benzene	Naphthalene	Ethylbenzene	Benzene
	W–MeOH			W–ACN		
	z_{SP} (nm)					
20	2.275	2.275	2.425	2.425	2.525	2.475
30	2.475	2.425	2.425	2.575	2.475	2.475
40	2.675	2.525	2.475	2.625	2.575	2.575
50	2.825	2.575	2.375	2.575	2.575	2.525
60	2.725	2.525	2.525	2.575	2.525	2.525
70	2.525	2.575	2.525	2.625	2.675	2.575
80	2.625	2.675	2.525	2.625	2.625	2.575
90	2.625	2.675	2.575	2.625	2.625	2.575

Table S3. Location of the stationary-phase limit, z_{SP}, for polar analytes at a given OS volume fraction in the W–MeOH or W–ACN mobile phase.

Vol % OS	Acetophenone (W–MeOH)	Benzyl alcohol (W–MeOH)	Phenol (W–MeOH)	Acetophenone (W–ACN)	Benzyl alcohol (W–ACN)	Phenol (W–ACN)
	z_{SP} (nm)					
10	2.375	2.375	2.325	2.425	2.425	2.375
20	2.375	2.375	2.375	2.475	2.475	2.375
30	2.425	2.425	2.375	2.475	2.475	2.425
40	2.425	2.475	2.425	2.525	2.525	2.475
50	2.475	2.475	2.475	2.475	2.475	2.475
60	2.525	2.425	2.525	2.525	2.525	2.475
70	2.525	2.525	2.525	2.625	2.525	2.525
80	2.525	2.475	2.525	2.625	2.525	2.525
90	2.575	2.525	2.575	2.575	2.525	2.525

Table S4. Solute-specific cutoff distances between the indicated solute and solvent atoms for determination of solute–solvent hydrogen bonds with W–MeOH and W–ACN mobile phases (top and bottom, respectively).

	W(H)	W(O)	MeOH(H)	MeOH(O)	ACN(N)
			r_{HB} (nm) [a]		
Acetophenone(O)	0.25	0.33	0.26	0.32	–
Benzyl alcohol(O)	0.26	0.35	0.26	0.35	–
Benzyl alcohol(H)	–	0.27	–	0.27	–
Phenol(O)	0.26	0.36	0.26	0.34	–
Phenol(H)	–	0.26	–	0.26	–
Acetophenone(O)	0.25	0.33	–	–	–
Benzyl alcohol(O)	0.26	0.35	–	–	0.34
Benzyl alcohol(H)	–	0.26	–	–	0.28
Phenol(O)	0.26	0.37	–	–	0.33
Phenol(H)	–	0.26	–	–	0.27

[a] Available from ref. 2 in the main text.

Figure S1. Orientation of polar analyte molecules in the partitioning peak as a function of the OS volume fraction in the W–MeOH (left) or W–ACN mobile phase (right). The angle φ is formed between the indicated molecular vector and the surface normal.

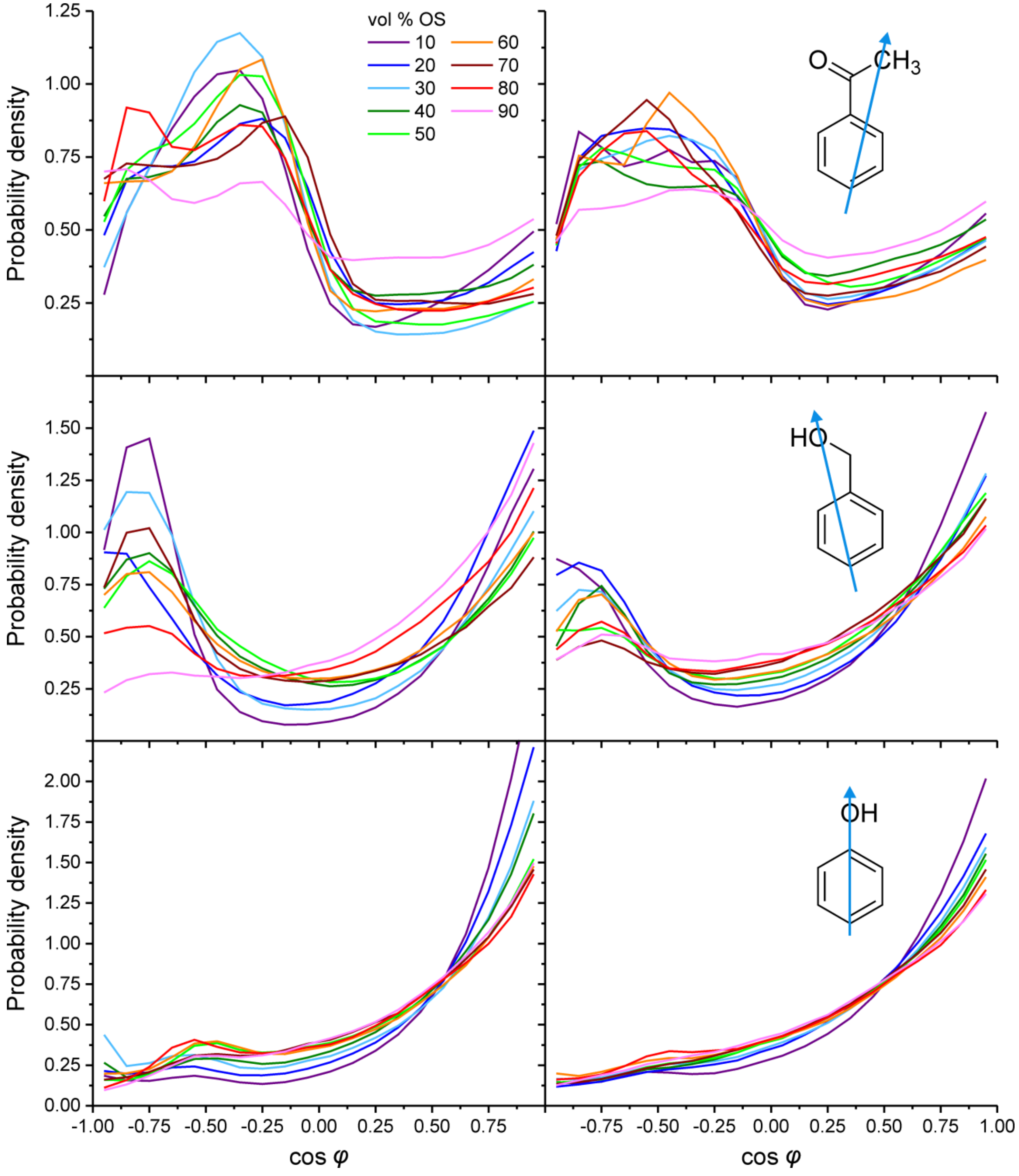

Figure S2. Orientation of polar analyte molecules in the adsorption peak as a function of the OS volume fraction in the W–MeOH (left) or W–ACN mobile phase (right). The angle φ is formed between the indicated molecular vector and the surface normal.

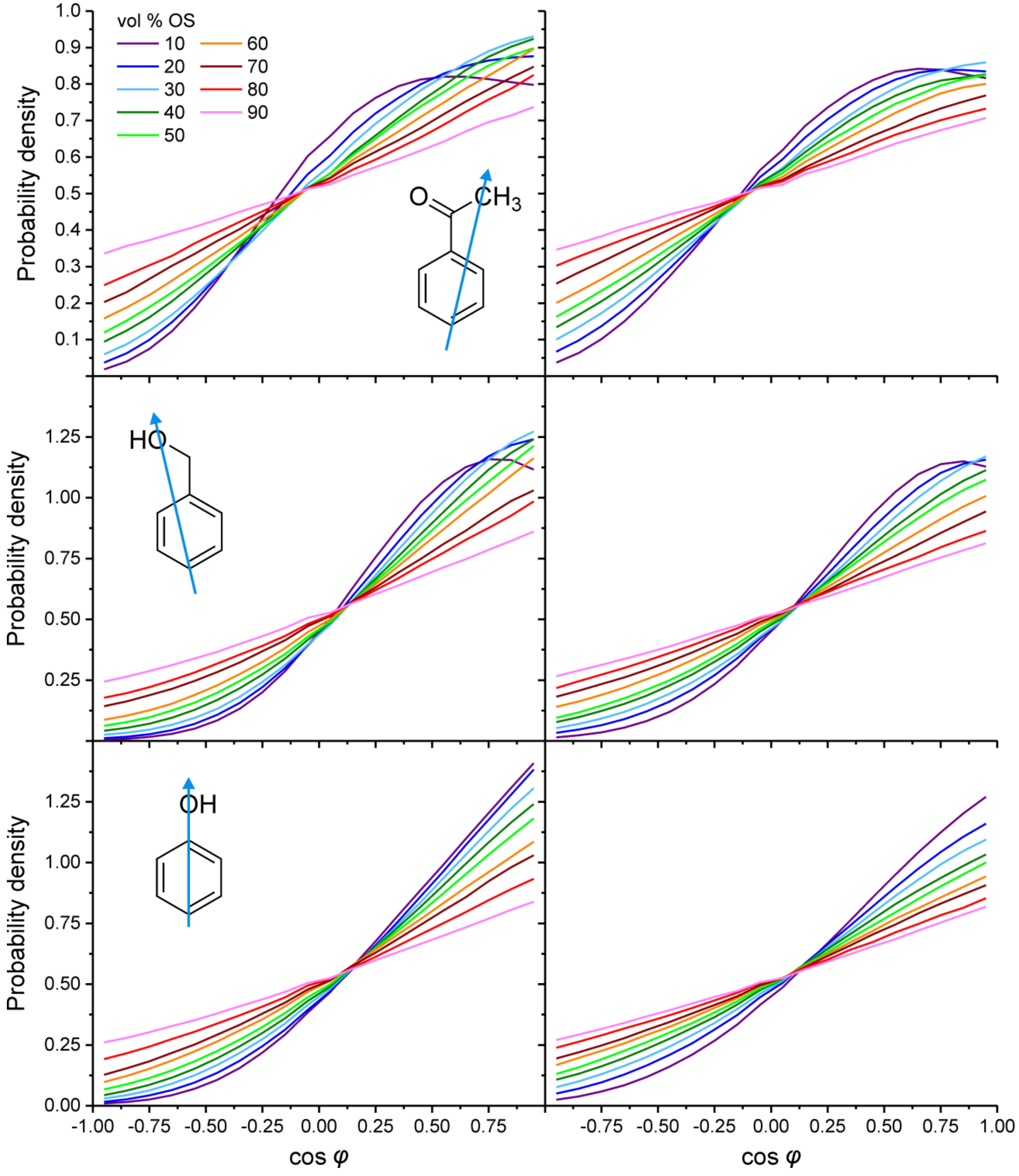

Figure S3. Orientation of apolar analyte molecules in the partitioning peak as a function of the OS volume fraction in the W–MeOH (left) or W–ACN mobile phase (right). The angle φ is formed between the indicated molecular vector and the surface normal.

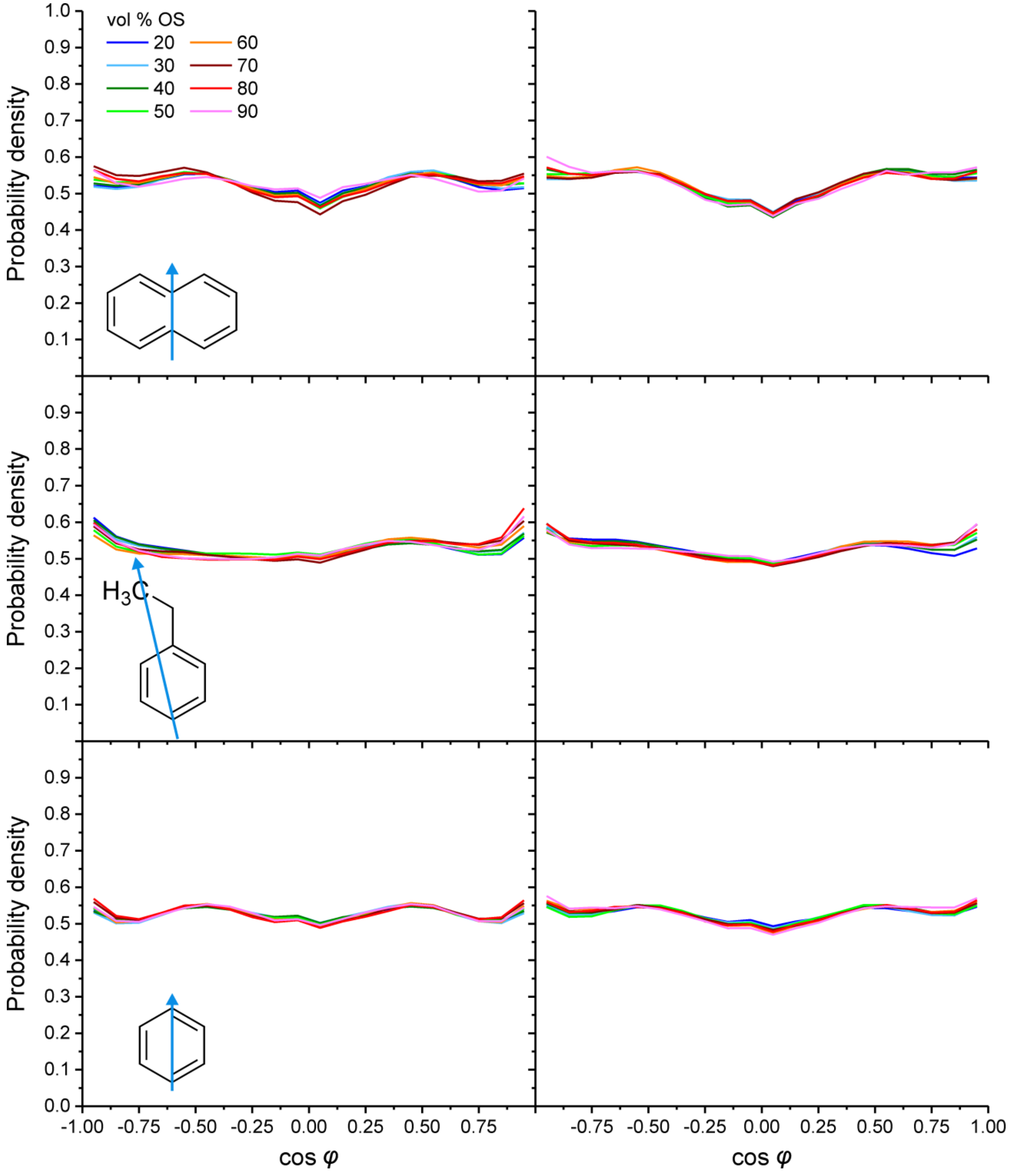

Figure S4. Orientation of apolar analyte molecules in the adsorption peak as a function of the OS volume fraction in the W–MeOH (left) or W–ACN mobile phase (right). The angle φ is formed between the indicated molecular vector and the surface normal.

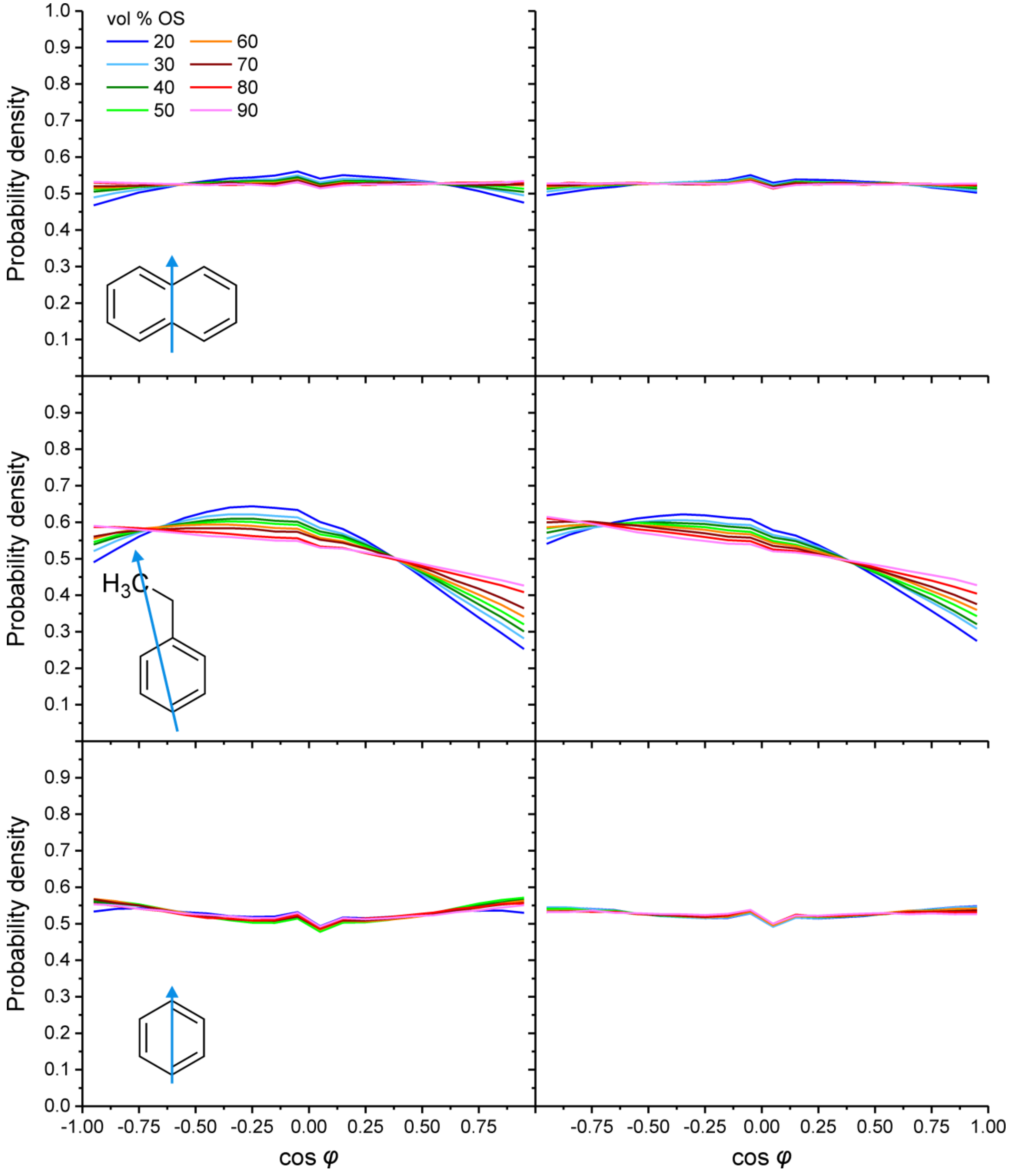

Figure S5. W hydrogen-bond partner density profiles for polar analyte molecules (acetophenone, benzyl alcohol, and phenol from top to bottom) in the adsorption peak (with snapshots visualizing the preferential solute orientation) as a function of the OS volume fraction in the W–MeOH (left) or W–ACN mobile phase (right).

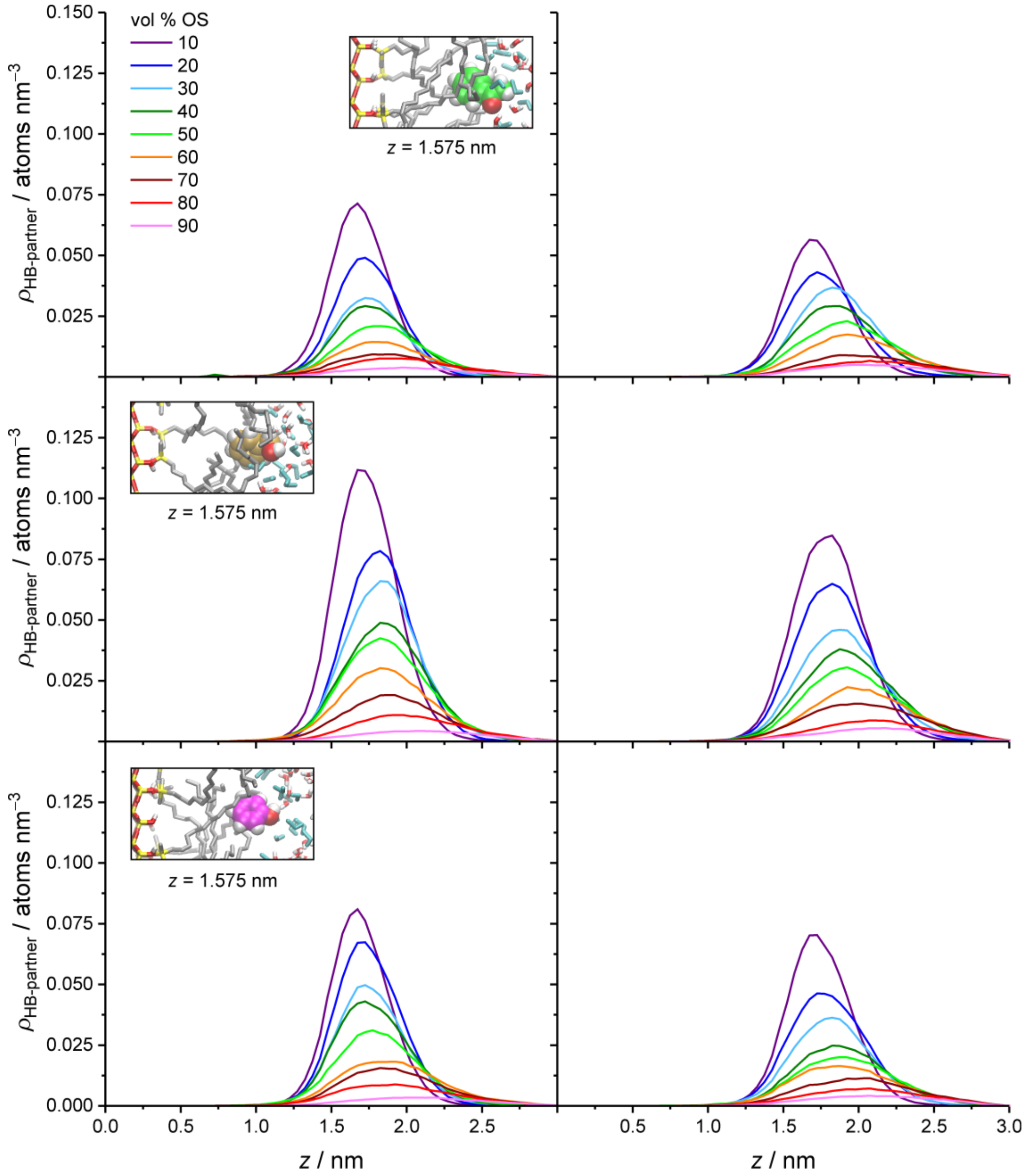

Figure S6. OS hydrogen-bond partner density profiles for polar analyte molecules (acetophenone, benzyl alcohol, and phenol from top to bottom) in the adsorption peak (with snapshots visualizing the preferential solute orientation) as a function of the OS volume fraction in the W–MeOH (left) or W–ACN mobile phase (right).

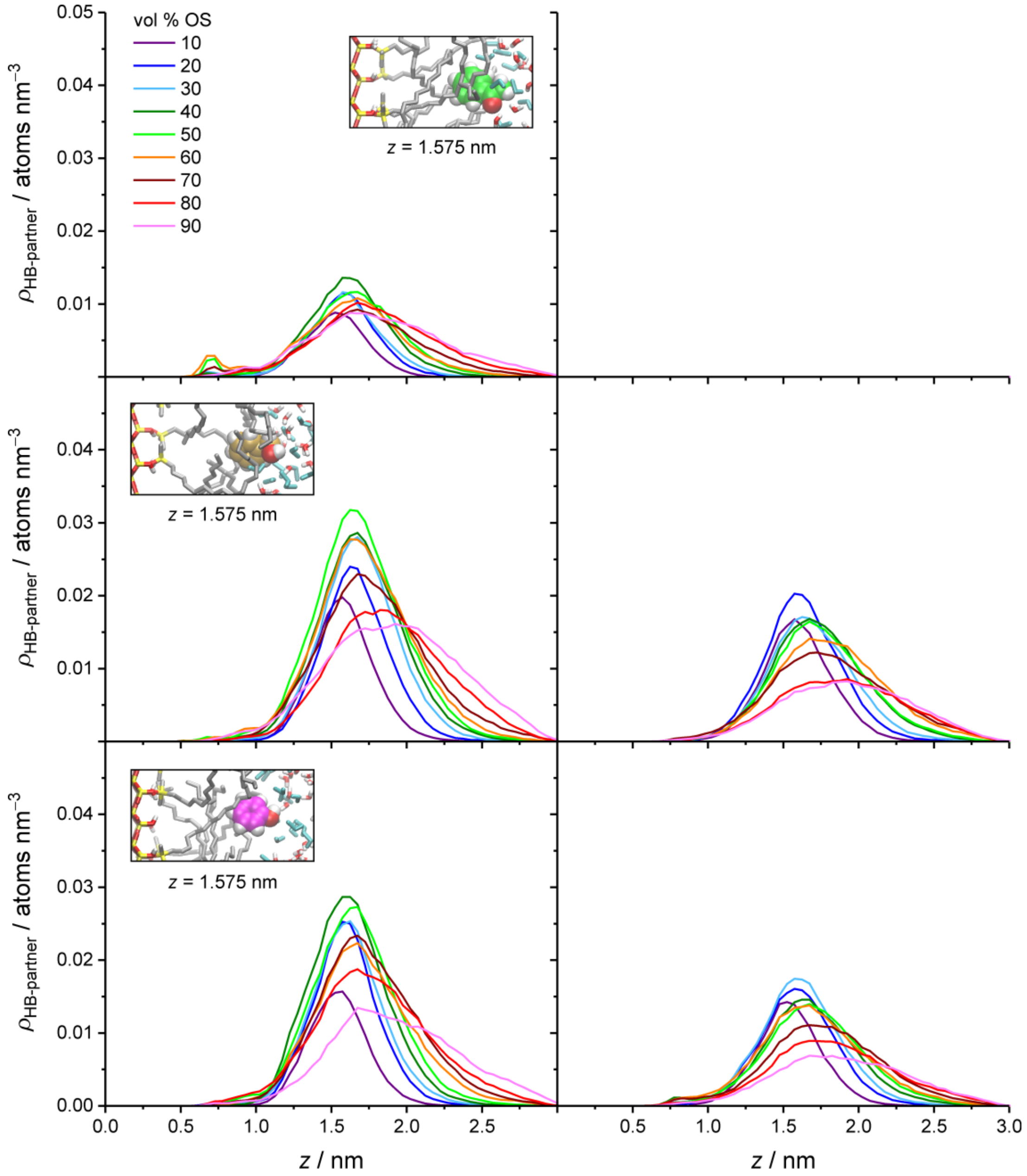

Supplement: Supplementary file 1 [file jp5c01697_si_001.pdf]
